# Using the Gibbs Function as a Measure of Human Brain Development Trends from Fetal Stage to Advanced Age

**DOI:** 10.3390/ijms21031116

**Published:** 2020-02-07

**Authors:** Edward A. Rietman, Sophie Taylor, Hava T. Siegelmann, Marco A. Deriu, Marco Cavaglia, Jack A. Tuszynski

**Affiliations:** 1 BINDS Lab, School of Computer Science, University of Massachusetts Amherst, 140 Governors Drive, Amherst, MA 01003-9264, USA; erietman@gmail.com (E.A.R.); hava@cs.umass.edu (H.T.S.); 2 Department of Physics, University of Alberta, 4-181 CCIS, Edmonton, AB T6G 2E1, Canada; trageser@ualberta.ca; 3 DIMEAS, Politecnico di Torino, Corso Duca degli Abruzzi 24, 10129 Torino, Italy; deriu.marco@gmail.com (M.A.D.); marco.cavaglia@fastwebnet.it (M.C.); 4 ACTISMED Research, srl, via Genova 4, 10126 Torino, Italy

**Keywords:** brain development, Gibbs free energy, thermodynamics, transcriptome, protein–protein interactions

## Abstract

We propose to use a Gibbs free energy function as a measure of the human brain development. We adopt this approach to the development of the human brain over the human lifespan: from a prenatal stage to advanced age. We used proteomic expression data with the Gibbs free energy to quantify human brain’s protein–protein interaction networks. The data, obtained from BioGRID, comprised tissue samples from the 16 main brain areas, at different ages, of 57 post-mortem human brains. We found a consistent functional dependence of the Gibbs free energies on age for most of the areas and both sexes. A significant upward trend in the Gibbs function was found during the fetal stages, which is followed by a sharp drop at birth with a subsequent period of relative stability and a final upward trend toward advanced age. We interpret these data in terms of structure formation followed by its stabilization and eventual deterioration. Furthermore, gender data analysis has uncovered the existence of functional differences, showing male Gibbs function values lower than female at prenatal and neonatal ages, which become higher at ages 8 to 40 and finally converging at late adulthood with the corresponding female Gibbs functions.

## 1. Introduction

Mathematical modelling and computational simulations have become indispensable tools in all areas of biological and biomedical research. Theoretical and experimental investigations of biological system dynamics involve mathematicians, computer engineers, physicists, and biologists who contribute collectively to the field in order to provide quantitative analysis and interpretation of complex bio-medical data. If correctly framed, molecular models provide an essential tool for studying complex molecular interactions. Multi-scale models of complex networks provide new insights into biological mechanisms integrating molecular, cellular, tissue, organ and potentially whole organism levels. In this setting, the human brain can be viewed as a system of unparalleled complexity, which is constrained by a fixed inflow of free energy in the form of biochemically-derived nutritional energy, principally in terms of ATP generated from glucose metabolism. Human brain’s neurodevelopment is importantly related to evolution, sexual dimorphism, and increased susceptibility to certain brain disorders. Comprehensive knowledge about the spatio-temporal dynamics of the brain’s transcriptome is important for a better understanding of molecular mechanisms involved. A major part of the transformations involved in the development of the human brain from prenatal to infant, to mature, and to advanced-age stages is metabolic demand-driven by both structure formation and functional needs. In this paper, we focus on structural evolution as reflected by the expression and utilization of proteins, which form complexes and interaction networks. This structural organization is reflected in the complexity of the corresponding protein–protein interaction (PPI) networks, which can be quantified using systems biology methods as we describe below.

## 2. Brain Development and Its Evolution

Brain development can be investigated from different perspectives: functional, metabolic or structural. Metabolism-based studies of the human brain using different methods converge to a common conclusion, namely, that the brain energy use, whether measured per unit of mass or for the brain as a whole, reaches its peak during childhood, not at birth [1,2,3,4]. This is true whether one uses 18F-fluordeoxyglucose positron emission tomography (FDG-PET), cerebral metabolic rate of glucose (CMRglucose), the nitrous oxide method or MRI methods that estimate oxygen use (CMRO_2_) or cerebral blood flow (or perfusion—as a per-gram measure). All these investigations and their outcomes indicate that the energy demands of the brain peak out in childhood (around 4–5 years), not at birth [5]. This has little to do with brain growth, which is nearly complete at this age, but instead relates to such measures as synaptic density, which peak out at this age. It is important in this connection to stress that the metabolic investigations of the brain development focus on the function of the brain that is directly related to metabolic demand. In this paper, we will revisit this issue from a protein-level standpoint and demonstrate that the peak of network-formation process does not coincide with the peak of metabolic demand but occurs earlier.

Human brain’s development is a complex and precisely regulated process that unfolds over a protracted period of time from prenatal stages to maturity leading to the organization of an immensely complex organ composed of hundreds of billions of cells (including approximately 100 billion neurons and perhaps ten times as many glial cells) [6]. Neurons are highly interconnected with an average of 10,000 synaptic connections to each neuron and in some cases up to 100,000 [6]. Neurons and glia, similarly to other eukaryotic cells, contain sets of widely distributed, specialized, and discretely localized proteins forming complexes and networks. A detailed documentation of global gene expression is now published in online repositories and more than 35,000 data sets offer links to human brain. Transcriptome studies conducted on human brain proteome have interestingly identified that the highest numbers of tissue-enriched genes are expressed in testis and in brain [7]. This finding supports the hypothesis that organ functions and properties are determined by the expression of proteins with specific molecular characteristics and cellular functions. Protein expression data are largely indicative of the formation of structure as well as its later degradation, not necessarily of the intensity of the organ’s functional engagement, which is reflected more directly by metabolic processes. Protein expression may, on the other hand, be indirectly indicative of longer-term structural changes such as synaptic plasticity and remodelling of the synaptic connections. In this paper, our interest lies in the investigation of how protein expression data can be used to measure the associated development of the brain’s structural organization from prenatal stages to advanced age. We will also address the question of differences in these datasets between male and female brains across the lifespan.

From a thermodynamic perspective, the transcriptome and other omic (e.g., proteomic, genomic, metabolomic, etc.) measures can represent the energetic state of a living cell, or indeed an organism as a whole, in terms of a non-equilibrium state of a living system [8]. The method we propose here demonstrates that the thermodynamic spatio-temporal profile of the human brain transcriptome, when compared with biological data from neurobiology literature, correlates from the DNA level all the way to the organ level. Our choice of the thermodynamic measure of the brain’s development, which may be viewed as entropy reduction associated with living processes, is the Gibbs free energy, which is relatively straight-forward to compute from protein expression data.

The network’s Gibbs free energy has origins in statistical physics where its increase signifies departure from a thermodynamic equilibrium. Conversely, a tendency of the thermodynamic system to attain the lowest Gibbs free energy under existing external constraints (e.g., constant pressure) is a consequence of the second law of thermodynamics and is consistent with a maximum entropy principle. The maximum entropy principle has been shown to be a very powerful organizing concept across all fields of science from physics to evolutionary biology to drug action and even human society [9,10]. As will be shown below, the most important findings of our work are that the human brain’s transcriptome has the lowest (most negative) Gibbs free energy values pre-birth, followed by a dramatic climb to the maximum Gibbs free energy at birth, which mirrors not only the growth of the organ itself but also the development of its complex organizational architecture. This rapid ascent to a maximum value is then followed by a gradual drop in the Gibbs free energy function to a local minimum around the age of sexual maturity, which could signal structural and organization stabilization of this organ. After that, another change in the trend occurs with a slight increase in the Gibbs energy function continuing into advanced age, which might be hypothesized to signify destabilizing processes of accumulated damage and structural deterioration. These pronounced trends, which are consistent across the various areas of the brain investigated here, separated by rather sharp transitions, are in our opinion related to important biological and physiological processes such as structure formation, building of neuronal connections, and also structural and functional deterioration due to aging along long-term trend timelines. We stress here that these results are obtained at the level of protein–protein interactions but are extrapolated to brain areas and the whole brain per se. Although we use the thermodynamic function of state, namely the Gibbs free energy, it is computed for protein concentrations, which has been applied elsewhere in systems biology as we discuss next. Interestingly, there are also documented actual thermodynamic transitions taking place in the human brain such as the sudden temperature change at birth, which corresponds to a change of conditions from a thermodynamically closed system to an open one [11,12]. The passage to old age is also known to correspond to volumetric changes of the brain as well as pathological protein aggregation such as seen in the formation of amyloid plaques [13]. These processes may be correlated with transformations at a protein network level but we have not found direct empirical evidence for this yet. Therefore, future studies in this direction would be of enormous value.

## 3. Systems Biology Methods

The theoretical underpinnings for the thermodynamics-inspired approach we propose to use in the present context in order to better understand the molecular biology of human brain development were developed over a several-year period focusing initially on disease initiation and progression issues. This previous work involved different biological examples including several types of cancer [14,15,16,17,18,19,20]. Here, we give a brief summary of this body of work to set the stage for the present case. The transcriptome and other -omic (e.g., proteomic, genomic, etc.) measures can be viewed as representing the thermodynamic state of a cell from the viewpoint of structural transformation, not necessarily metabolic activity. It is well known that every living system is out of thermodynamic equilibrium simply because of a constant need for metabolic energy production. This is a major difference between animate and inanimate matter as eloquently explored by E. Schrödinger in his seminal book entitled ‘What is Life” [21]. A living cell uses nutrients such as glucose and transforms them into ATP as the universal biological energy currency required for structure formation and biological function. One of the main energetic demands of every cell is the production of specific proteins, which are used for numerous structural and functional needs of a cell. Protein expression levels, therefore, can be viewed to represent a proxy measure of the living cell’s non-equilibrium thermodynamic energy state. Moreover, proteins interact with other proteins generating very complex protein–protein interaction networks whose architecture is cell-specific. There is a chemical potential between interacting molecules in a cell, and the chemical potential of all the proteins that interact with each other can be viewed as forming a rugged landscape, not dissimilar to Waddington’s epigenetic landscape [22,23]. The above formulates our conceptual framework for the foregoing analysis. There have been recent advances in using network analyses to represent both the nervous system and neurological disorders, but they were typically performed at the level of image deconstruction at the whole brain level [24]. An excellent overview of the connections between hierarchical scales of brain organization using network representations can be found in Reference [25].

**Figure 1:**
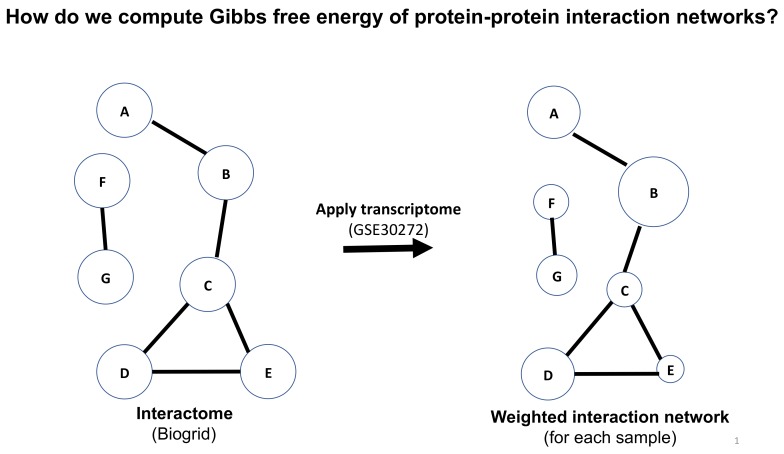
A schematic illustration of the process of computing the Gibbs free energy for a protein–protein interaction network.

The method we propose uses mRNA transcriptome data or RNA-seq data as a surrogate for protein concentration values. This assumption is largely valid. In fact, References [26,27] have shown an 83% correlation between mass spectrometry proteomic information and transcriptomic information for multiple tissue types. Further, Reference [28] found a Spearman correlation of 0.8 in comparing RNAseq and mRNA transcriptome from TCGA human cancer data [29]. Given the set of transcriptome data, a representative of protein concentration, we overlay that on the human PPI network from BioGrid [30]. This means we assign to each protein (representing a node with edges assigned to its interactions with other proteins, or nodes) in the network, the transcriptome value (or RNAseq value) after rescaling. From that we then compute the Gibbs free energy of each PPI using the standard statistical thermodynamic relationship (see Equation (1) below), which employs protein concentration values, i.e., *c_i_* is the “concentration” of the protein *i*, normalized, or rescaled, to be between 0 and 1 corresponding to minimum and maximum values, respectively. The sum in the denominator is taken over all protein neighbors of *i*, and including *i*. Therefore, the denominator can be considered akin to degree-entropy as pointed out elsewhere [16,17,18,19,20]. Carrying out this mathematical operation essentially transforms the “protein concentration” value assigned to each protein to its Gibbs free energy contribution. Thus, we replace the scalar value of transcriptome to a scalar function—the Gibbs free energy (see Figure 1). As we mentioned above, this strategy has been used previously in other contexts to uncover trends shown by large datasets such as cancer signaling proteins and to find out how to measure evolutionary tendencies, for example disease progression over time. Here, we apply this methodology to quantify large data sets that have been generated for human brain proteins as described below.

## 4. Data Sources and Data Analysis

For our analysis, we used a data set representing post-mortem samples of brain tissue from human subjects. We downloaded the data sets from GEO using the GPL5175 platform (Affymetrix Human Exon 1.0 ST Array): GSE30272 [31], GSE18069 [32], and GSE25219 [33,34], as well as BrainSpan [35,36]. Only GSE25219 was selected for further analysis. GSE25219 contained 57 subjects, 16 brain regions, L and R hemispheres (for only 39 of the subjects), which included 31 males and 26 females. The samples obtained were from deceased donors presenting without a clinical brain pathology or signs of serious genomic abnormalities [33]. We then combined soft matrix data with gene expression data files. IDs in the gene expression data were matched to gene names (several ID numbers can be matched to a single gene name). Where there were duplicate entries for a gene, the average of the expression values was taken. If there was no gene name that could be matched to an ID, the entry was deleted. The output file created contains a list of genes (rows) and samples (1340 columns) with the averaged log2 expression values for each entry. We then calculated Gibbs free energy values associated with each sample and plotted the results (see below) by binning data into age groups. The age ranges were determined based on natural breaks in the data set, developmental stage, and considerations based on the amount of data in a given set (for example, a bin might be increased if there was insufficient data within the bin). The difference in binning ranges between plotting sets (i.e., sex, protein, overall, hemisphere) is due to this third factor. Error bars are based on standard error calculations. Some of the fetal regions used a different labelling system. In our analysis, any data that were labelled using a label that could not be directly matched to one of the 16 brain regions mapped were discarded. Fortunately, this constituted a very small fraction. All of the final plots were produced using the R software, along with the accompanying statistical data.

As mentioned above, the method we applied here used mRNA transcriptome data or RNA-Seq data as a surrogate for actual protein concentration. This assumption is largely valid. Kim et al. [37] and Wilhelm et al. [38] have shown an 83% correlation between mass spectrometry proteomic information and transcriptomic information for multiple tissue types. Further, Guo et al. [39] found a Spearman correlation of 0.8 in comparing RNA-Seq and mRNA transcriptome from TCGA human cancer data [29]. We believe this justifies the use of this methodological simplification, especially since we are interested in trends and not in the quantification of minute differences.

Given the set of transcriptome data, a representative of protein concentration, we then overlaid that on the human PPI network from BioGRID [30,40]. This means we assigned to each protein in the network, its scaled (between 0 and 1), transcriptome value (or RNA-Seq value). From that we computed the corresponding Gibbs free energy function for each protein taking into account its protein–protein interactions using the relationship [16,20]:

(1) $$G_{i}=c_{i}\;ln[\frac{{}_{i}c_{i}}{\Sigma_{j}\;c_{j}}]$$

where *c_i_* is the “concentration” of the protein *i*, normalized, or rescaled, to be between 0 and 1. The sum in the denominator is taken over all protein neighbors of *i,* and including *i.* Therefore, the denominator can be considered related to a degree-entropy of the underlying PPI network. Carrying out this mathematical operation essentially transforms the “concentration” value assigned to each protein to a Gibbs free energy contribution, which can eventually be added up for the entire set of expressed proteins providing an overall thermodynamic measure for the state of the system at a given point in time corresponding to the time when the tissue sample was obtained. Thus, we replace the set of scalar values associated with a transcriptome by a scalar function—the Gibbs free energy function. By summing over the whole PPI network, we can compute the Gibbs free energy function (Figure 1), which represents the energetic state of the autopsied tissue sample and hence provides a measure of a thermodynamic state of the donor’s brain at the time of death.

The transcriptome data provide quantitative information about the mRNA expression levels in a given sample. Expression values are log2 normalized. These expression levels, *e_i_*, act as a proxy for protein concentration. The concentrations values, *c_i_*, are further normalized into the interval <0,1> where a value of 1 suggests a high relative protein concentration, *e_max_*, while a value of 0 corresponds to a low relative protein concentration, *e_min_*, according to:

(2) $$c_{i}=\;\frac{e_{i}-e_{min}}{e_{max}-e_{min}}$$

With these two sets in hand, we superimpose the protein concentration data on the PPI. A chemical potential is then assigned to each node using the relationship:

(3) $$\mu_{i}=\ln\left[\frac{c_{i}}{\sum_{j\in Adj\left(i\right)\cup \left\{i\right\}}c_{j}}\right]$$

where the denominator is the sum of the concentrations of adjacent nodes. The term in the argument of the natural logarithm function will be equal to 1 if the protein is unconnected (or all adjacent nodes have 0-concentraction values). Generally, we can see that $\mu_{i}$ will be greater for higher values of *c_i_* and lower concentrations in adjacent nodes. The magnitude of the potential is therefore greater for low concentration proteins with highly upregulated adjacent nodes.

**Figure 2:**
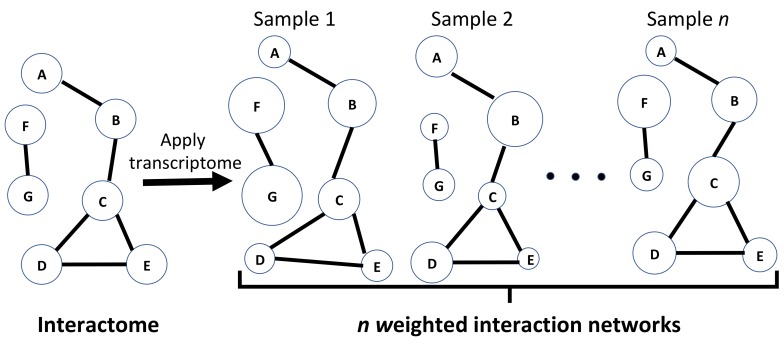
A schematic illustration of the method to calculate the average values of the Gibbs free energy over a number of samples. Here, n represents the number of samples used.

A chemical potential can be associated with the proteins that interact with one another that can be viewed to represent a rugged landscape [41,42]. In particular, the human brain may be regarded as a thermodynamic system out of equilibrium that is thermally shielded from the environment by maintaining a constant temperature and pressure. Its internal dynamical processes have been earlier described elsewhere using thermodynamics concepts as representing a path on the saddle surface of the entropy production rate [43]. Our computations provide snapshots of thermodynamic states quantitated by protein expression values, which, when combined, give the Gibbs free energy function. If these snapshots could be generated systematically over a period of time, one could see a time-development trajectory of an individual brain’s history. Without access to such data, we sampled the snapshot values obtained from the individual brains at their time of death and analyzed each region of the brain separately. Knowing the age of each subject allowed us to generate an age-dependent function for the Gibbs free energy by calculating average values of the Gibbs free energy function for a set of donors within the same age bracket and with the same sex (see Figure 2). This allowed us to obtain a comparison between different age groups and between female and male brains as is discussed below.

To this end we have performed a simple calculation to obtain the total Gibbs free energy for the network:

(4) $$G=\;\sum_{i}c_{i}\mu_{i}=\;\sum_{i}c_{i}\ln\left[\frac{c_{i}}{\sum_{j\in Adj\left(i\right)\cup \left\{i\right\}}c_{j}}\right].$$

We performed these calculations for several transcriptome data sets, however the focus of this paper is specifically on the data set GSE25219 [33,34] from the NCBI GEO database [44,45] which contains 1,340 samples obtained from 57 healthy post-mortem human brains. This data set contains quantitative information for 16 brain regions with samples including both sexes and multiple age groups ranging from prenatal to advanced age adult [33]. A list of these brain regions, their acronyms used, and their location can be found in Table 1 [33]. The PPI data were obtained from BioGRID [30,40]. The Gibbs energy was computed using Python 2.7. Biological interpretation of thermodynamic findings has been carried out by reviewing the relevant scientific literature.

Before we present the results of this analysis, we briefly outline the workflow involved in the data analysis. We downloaded the data from the following data sets from GEO using the GPL5175 platform (Affymetrix Human Exon 1.0 ST Array): GSE30272 [31], GSE18069 [32], and GSE25219 [33,34], as well as BrainSpan [35,36]. Only GSE25219 was selected for further analysis. GSE25219 contained 57 subjects, 16 brain regions, L and R hemispheres (for only 39 of the subjects), 31 males, and 26 females. The samples obtained were from normal donors without a clinical brain pathology or signs of serious genomic abnormalities [33]. We then combined soft matrix data with gene expression data files. IDs in the gene expression data were matched to gene names (several ID numbers can match to a single gene name). Where there were duplicate entries for a gene, the average of the expression values was taken. If there was no gene name that could be matched to an ID, the entry was deleted. The output file created contains a list of genes (rows) and samples (1340 columns) with the averaged log2 expression values for each entry. We then calculated Gibbs free energy values associated with each sample and plotted the results (see below) by binning data into age groups. The age ranges were determined based on natural breaks in the data set, developmental stage, and considerations based on the amount of data in a given set (for example, a bin might be increased if there was insufficient data within the bin). The difference in binning ranges between plotting sets (i.e., sex, protein, overall, hemisphere) is due to this third factor. Error bars are based on standard error calculations. Some of the fetal regions used a different labelling system. In our analysis, any data that were labelled using a label that could not be directly matched to one of the 16 brain regions mapped were discarded. Fortunately, this constituted a very small fraction. All of the final plots were produced using the R software, along with the accompanying statistical data.

## 5. Analysis of the Results

**Figure 3:**
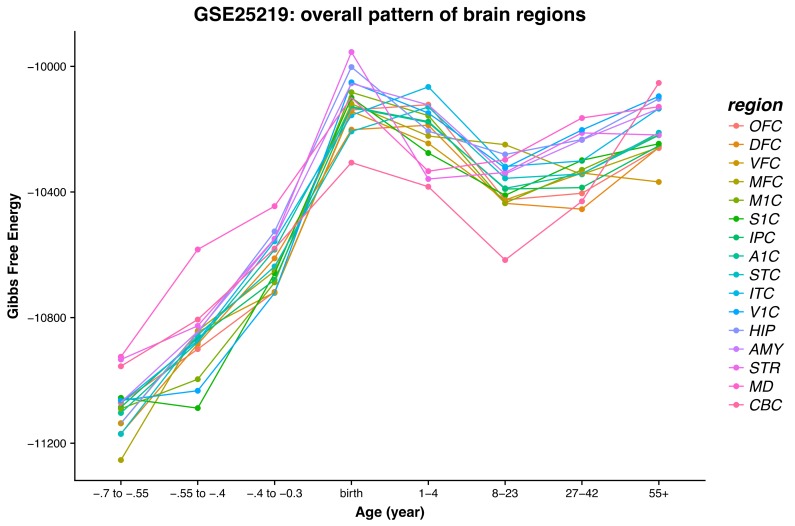
Plot of the Gibbs free energy for the 16 main brain regions averaged over the individual data sets and binned according to age groups.

In this section we provide an overall transcriptome time dependence of the corresponding Gibbs free energy and a discussion of the physiological interpretation when available. Figure 3 shows the overall patterns in the 16 regions of the human brain from prenatal stages to advanced age. In Figure 3, a simple average has been taken for all samples of a particular brain region for a given age range (with suppressed hemisphere and sex information). We can observe a consistent upward trend in the fetal period across all brain regions. The three regions that do not appear to increase between the first and second time periods are V1C, S1C, and M1C, and the upper outlier in the second bin is MD. The data are in accordance with the embryologic development time of V1C, S1C, and M1C not yet present [46]. The high values of the Gibbs free energy for MD, on the contrary, are related to its earlier and complex development, starting immediately after the formation of the neural tube [47,48]. The data show a consistent upward trend of the Gibbs free energy throughout the fetal development period, across all brain regions, culminating at birth except for the Occipital cortex areas V1C, OFC, A1C, and DFC that reach the highest Gibbs free energy at a 1–4-year point. This can be explained as the combination of dynamic cellular processes (glial mitosis versus pruning) occurring while cortexes are still developing and reach completion at around 1–2 years of age [46,49,50]. We can associate an increase in the Gibbs free energy with a thermodynamic tendency to move out of equilibrium, which is characteristic of growth and form generation. Later in the development, a dynamic equilibrium sets in whereby mitosis starts being balanced by apoptotic events that energetically stabilize the system [51] leading to a Gibbs free energy plateau.

At the beginning of the fetal age, the data reveal the overall lowest Gibbs free energy values, which steadily climb to a global maximum around the time of birth. This highly dynamic process taking place during the fetal development is probably due to the high rate of mitosis and physical expansion of the brain [52]. Note that the Gibbs free energies are calculated based on the fetus brain specimens, ranging between 2.3 and 5.4 cm of total length [48]. We hypothesize that the well-known sudden drop of the child’s body temperature at birth may be consistent with the findings of the Gibbs free energy decline and could also be responsible for reprogramming of the neuronal DNA interaction network. Change in gene interaction network levels has been demonstrated to be related to either an increase or decrease in entropy levels [13]. Furthermore, sudden temperature change at birth appears to be the most important thermodynamic signal to neuronal DNA [11,12,53], hence thermodynamics does play a role at this stage.

After birth we generally observe that a Gibbs free energy drop-off is followed by a levelling off or a slight upward trend. The data in this range are more variable between the brain regions relative to the fetal stages. The most significant outlier we can observe is CBC, which has the lowest Gibbs free energy from childhood through adolescence, and then, it jumps to the highest Gibbs free energy in the 55+ range, which may be related to aging and perhaps even structural and functional deficits.

We now focus on the next stage in the development of the brain occurring from birth to the age of maturity, i.e., approximately 23 years. Data in this range are more variable between brain regions relative to the fetal stages. After birth, we can generally see a Gibbs free energy drop-off followed by a levelling-off trend extended throughout childhood to sexual maturity. The brain structural/volume change, occurring after birth, is thermodynamically oriented towards its ordered developmental completion [13,33].

In the age bracket of 8–23 years, all brain regions, except AMY and STR, show a steady decrease in their respective Gibbs free energy compared to their values at birth. This can be correlated with structural consolidation especially pronounced after reaching sexual maturity. The brain’s complete development could also send a signal to switch metabolic preferences between anabolic and catabolic energy production processes [8]. Amygdala and striatum are developmentally interconnected since they both originate from the arc shaped “striatal ridge” [22,23,26]. The singularity of the cerebellum’s structure and function has been demonstrated in the literature [27,28]. We see in our results (see Figure 3) that CBC reaches the lowest Gibbs free energy value in the age bracket from 8 to 23 years when cerebellum is known to reach its complete development. Immediately after, MRI studies show a marginal volume decline that at the age of 50 becomes exponential, which could explain our findings [54,55,56].

## 6. The Brain Development and Evolution Trajectory in Relation to Gender

In this section we specifically analyze the differences between the Gibbs free energy results obtained for the male and female brains, respectively. We discuss these differences for each area of the brain separately. The corresponding graphical information is presented as Supplementary Information.

For the primary auditory cortex A1C (see Figure S1), for each age group there is no difference between male and female subjects beyond statistical error. A significant increase in Gibbs free energy can be observed, peaking at birth and then dropping moderately until the adolescent age region (ages 8–23) is reached, and then increasing slightly again for the last two age brackets. Generally, changes appear more exaggerated for the female subjects (lower troughs and higher peaks in the Gibbs free energy values). A recent publication analyzing post-mortem brains showed gender differences in the cerebral organization of primary auditory cortex. In particular A1C was found to be significantly larger in women than in man bilaterally [57].

For the amygdala AMY (see Figure S2), the overall pattern is as described for A1C, above. The female regions are consistently greater than the male counterparts until birth at which point the Gibbs free energy values for female samples peak. The corresponding values for male samples peak in the next bracket (1–4), however there is a significant error associated with this data point. This is followed by a dip in the adolescent bracket and a slight increase after. Again, we see a crossover along the age axis between the male and female Gibbs free energy values; the male subjects are characterized by more stable Gibbs free energy profiles, particularly in the last three age brackets (8+). A neuroimaging study conducted on the growth trajectories for the amygdala have found a statistically-significant difference in volume growth between males and females. While both sexes experienced similar rates of volume growth in early childhood, this growth rate rapidly slowed in females around the age of 13. On the other hand, in males, the most rapid period of deceleration in growth occurred in their late 20s [58].

The data for the cerebellar cortex CBC (see Figure S3) seem to differ relative to many of the sex plots. Here, the fetal region does not have as significant of an upward trend. For the female subjects, there is a moderate upward trend and then a sudden peak at birth that immediately drops again after birth. The male region does not appear to have the typical linear upward slope; however, there is no data for the youngest range and a relatively large statistical error in the second range. At birth, the male samples show Gibbs free energies, which are significantly lower than those for the females. After birth, the male and female samples effectively move in unison with no statistical differences. The dip and upward swing after age 8 are more pronounced for the cerebellar cortex. Using anatomic brain MRI scans from 25 healthy females and 25 healthy males aged 5–24 years, it was discovered that the total cerebellum volume followed an inverted U shaped developmental trajectory peaking at age 11.8 years in females and 15.6 years in males. Cerebellar volume was 10% to 13% larger in males depending on the age of comparison [54].

The data for the dorsolateral prefrontal cortex DFC seems to fall somewhere between the AMY and A1C, and CBC data (see Figure S4). Generally, there is an upward trend in the Gibbs free energy from the inception of the fetal stage until birth. Similarly to the CBC data, the upward trend is not present between the second and third age bracket (covers −0.55 to −0.3) for the male subjects only. Again, the male subject Gibbs free energy peaks slightly over birth in the 1–4 bracket. Both sexes exhibit the 8+ dip and swing. As with A1C and AMY, this dip and swing is more pronounced for female subjects.

In the hippocampus HIP data (see Figure S5), we observe the same upward trends until birth. Overall, the data for the male and female regions appear quite similar. Both have a peak at birth (though more significant for the female region). Unlike other regions, there, the Gibbs free energy is more stable after birth, particularly for the male subjects. The female subjects again have an upward swing in the Gibbs free energy but mostly in the 40+ years region.

For the data for the posterior inferior parietal cortex IPC (see Figure S6), the upward trend is more modest in the fetal samples. The female samples have a sudden jump at birth, and then, the Gibbs free energy drops down abruptly (although not to fetal levels). The male subjects also show a large increase at birth but this continues into the 1–4 age bracket (however, there is significant error associated with this data point). The typical dip and swing is then observed as well as the sex crossover mentioned above.

For the fetal period for the inferior temporal cortex ITC (see Figure S7), we can observe an upward slope in the Gibbs free energy; however, its magnitude is greater for the female subjects. The female regions exhibit the typical peak at birth, followed by a dip and then increase again at age 40+. The male region has a clear peak in the 1–4 age range, then, it dips modestly and then remains relatively stable. Again, we can see a sex crossover effect after birth.

The Gibbs values for the primary motor cortex M1C (see Figure S8) are quite typical relative to other brain regions. There is an upward trend, peaking at birth for the female samples and peaking slightly in the 1–4 region for the male subjects. Both sexes move approximately in unison, dipping in the 8–23 bracket and then swing upward slightly thereafter.

The Gibbs values for the mediodorsal nucleus of the thalamus MD (see Figure S9) are somewhat atypical relative to other brain regions. On reason for this may be that there was less data for this region (in particular it is worth noting that there was only one male sample in the 1-4 age bracket). The female subjects follow an upward trend, peak at birth and then dip down after birth and remain relatively stable. The male curve is more sporadic, peaking at birth then dropping off abruptly and swing back up to approximately the same value observed at birth.

For the medial prefrontal cortex MFC (see Figure S10), we see a typical pattern in the female region: an increase during fetal development, a peak at birth immediately followed by a dip, and then a slight upward swing in the 40+ region. The male regions on the other hand, follow a similar fetal developmental pattern (translated down slightly), however the is no dip and swing. Instead we basically see a plateau after birth and a slight drop in the 40+ region.

The orbital prefrontal cortex OFC (see Figure S11) follows a pattern similar to the one observed in the ITC (see Figure S7). The difference here is that the jump between the third fetal period and birth is more significant than in the ITC. Like the ITC, the male region peaks after birth (however there is only one data point) and is stable after it dips down in the 8–23 range. The female curve follows the dip and swing and we also see the sex crossover pattern.

The Gibbs values for the primary somatosensory cortex S1C (see Figure S12) again display an upward trend during fetal development and a peak at birth for the female subjects, with the male values increasing slightly after birth. Both curves dip into the 8–23 age bracket; however, the male region has an anomalous peak again in the 27–40 age bracket. The female region remains relatively stable after the dip.

The posterior superior temporal cortex STC (see Figure S13) contains many of the patterns observed in the other brain regions. One thing that makes this region unique, is that it is the only one where the highest male value is greater than the largest female Gibbs energy. This may in part be due to the female peak being a bit less pronounced. Overall, we see the upward trend during fetal development (with a stall again between the second and third period for male samples), with peaks at birth and in the 1–4 age bracket for female and male samples, respectively. The female region has the dip and swing whereas the male samples are relatively flat in later years. The pattern also seems reminiscent of V1C (see Figure S15).

For the striatum STR (see Figure S14), we see somewhat erratic behaviour similar to that which was observed in the MD (see Figure S9). Like the MD, this may in part be due to fewer samples being available in this region (note, only one male sample in the −0.4 to −0.3 and 1–4 age brackets). The female region, like in the MD follows an upward trend until peaking at birth. The curve then dips down during the toddler years but then remains stable thereafter.

The female samples for the primary visual cortex V1C (see Figure S15) follow a typical pattern: upward slope during fetal development, peak at birth, dipping until 8–23 bracket, followed by an upward swing. The male pattern is similar to that observed in the S1C (see Figure S11): peaking after birth, then dipping in the 8–23 bracket and increasing again, plateauing from age 27 onwards. Overall, V1C and S1C appear quite similar.

**Figure 4:**
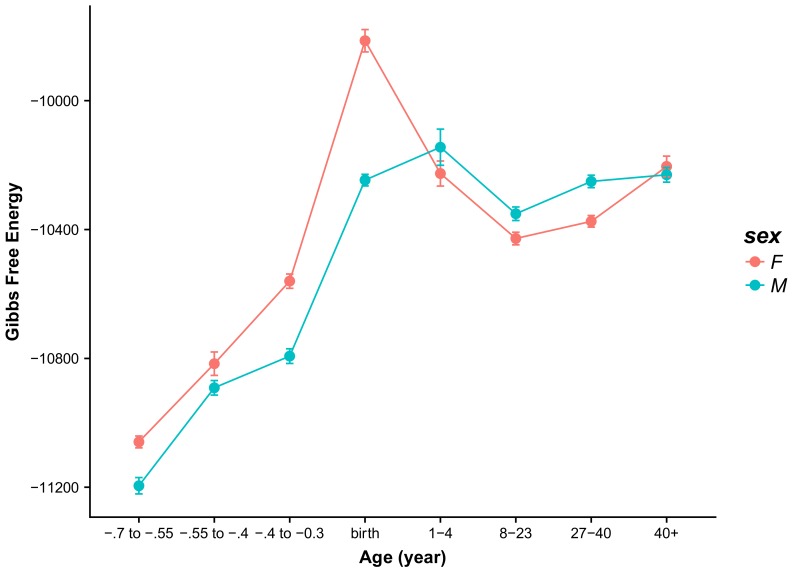
Plot of the Gibbs free energy values averaged over all 16 brain areas and presented for female and male cases separately.

Overall, there seems to be more stability in the ventrolateral prefrontal cortex VFC (see Figure S16) relative to other brain regions. The female samples show only a slight increase in the Gibbs free energy during fetal development and then a jump at birth. The Gibbs free energy drops linearly until the 8–23 bracket after which point the curve is effectively flat. For the male subjects, there is a step from the first fetal period to the second and then another step at birth, after which point the Gibbs free energy values remain relatively unchanged. To demonstrate that the cross-over effect is a general effect and not limited to specific regions of the brain, we have averaged the Gibbs free energy values over all brain regions for males and females separately and plotted the results in Figure 4. As can be readily seen, the cross-over effect in early childhood, around the age 1–4 years is consistently present. Moreover, in middle age, around the age of 40, both female and male brains converge to the same value of the Gibbs free energy.

## 7. Discussion

In this paper, we have analyzed protein expression data obtained from post-mortem brains of females and males spanning a range of ages from prenatal to advanced age. The data were organized according to their origin involving 16 distinct and well-defined brain regions. We have introduced a quantitative measure that reflects the structural brain development level based on the PPI network’s characteristics. This measure is the PPI network’s Gibbs free energy, which has its conceptual origins in statistical physics. We applied this measure to address the question if there are any trends that correlate with age and brain regions, and secondly, if there are differences in this regard between males and females. Our analysis shows a dramatic increase in the Gibbs free energy in prenatal brains as they approach the time of birth at which point the Gibbs free energy values consistently achieve a maximum value possibly reflecting the brain’s structural and functional development. Subsequently, the Gibbs free energy decreases with age achieving a local minimum around the age of sexual maturity. From that age onward, the Gibbs free energy slowly increases in almost all brain regions. Similar results, exploring functional entropy as a metric, were obtained by Yao et al. [13] who used image entropy values to characterize intrinsic ageing properties of the human brain from fMRI data sets, showing a trend of the functional entropy increasing with age. Note that entropy is inversely correlated with Gibbs free energy. Finally, we have also compared the Gibbs free energy for male and female samples, respectively. In most brain regions and also for the averaged values over all regions of the brain we found that from prenatal stages of development until early childhood the Gibbs free energy calculated for female subjects is higher than that of the male counterparts. According to literature the brain’s metabolic demands peak during childhood [1], which possibly signifies a different trajectory (accelerated earlier) in the development of female brains than is the case for male brains. A cross-over effect can be seen in early childhood where the relationship is reversed but eventually, around the age of 40, both curves converge to the same value. Admittedly, the relatively small sample size suggests that these results should be interpreted with great caution. Larger sets of similar data would offer greater confidence in our analysis but due to lack of such datasets we are unable to provide more statistically significant conclusions.

From a biological perspective, we know that 74% of the human transcriptome is over-expressed in the brain compared to other organs, meaning that brain harbors 14,518 out of all human proteins [59,60]. We are at the beginning of understanding how genes and neurons work together, akin to software and hardware, to make brain function possible. The study in Reference [1] represents the largest survey on adult human brain gene expression ever published to date and demonstrates that in the adult brain, some genes show reproducible expression patterns across neuroanatomical structures in different individuals. The importance of this finding is the demonstration that these stereotyped genes have a number of interesting correlations with neuronal functions, disease associations, and even drug targeting [61]. We find that the data corresponding to age/time-dependent shifts in the Gibbs free energy are remarkably similar across different brain regions, yet expression profiles and developmental changes differ strikingly from region to region. Cerebellum, for example, develops late compared with cerebral cortical structures, yet the time courses are nearly identical. Synaptic plasticity develops later in the cerebellum compared with other structures. Perhaps these differences can be understood at a higher organization scale than the PPI network scale used here. By far, the most interesting observations pertain to sex differences associated with generally lower Gibbs free energy in males during the prenatal and early childhood period. Later in the course of life, particularly in the post-adolescent period, these values are similar.

A previous study based on gene ontology and antibody-based tissue microarray analysis of the corresponding proteins found most brain-enriched protein coding genes to be found in astrocytes, oligodendrocytes, or in neurons with molecular properties linked to synaptic transmission and brain development [7]. Moreover, recent findings show that approximately 86% of protein-coding genes are differentially regulated at the whole transcript or exon level across regions and/or time, and most spatio-temporal differences occur before birth, followed by an increase in the similarity among regional transcriptomes during postnatal lifespan. These findings demonstrate that genes are organized into functionally distinct co-expression networks, and sex differences are present in gene expression and exon usage [33]. In recent years, biophysical studies have been providing thermodynamic interpretation of biological processes adding the necessary conceptual framework, which helps to navigate the inherent complexities and better understand biology without adding superfluous or misleading information [62].

Our aims in this work were to investigate if there are general trends or patterns in the human brain transcriptome expression across age groups and brain areas and if some trends could be gender related. Our paper represents the first application of a thermodynamic measure of human brain development, from fetal stage to advanced age adulthood, calculated as the Gibbs free energy values of PPI networks extracted from protein expression in all major brain regions. Data have been obtained from post-mortem brains of females and males spanning a range of ages from prenatal to advanced age adulthood. The data were organized according to their origin involving 16 distinct and well-defined brain regions. Our analysis shows a dramatic increase in the Gibbs free energy in prenatal brains as they approach the time of birth at which point the Gibbs free energy values consistently achieve a maximum value possibly reflecting the brain’s structural and functional development. Subsequently, the Gibbs free energy decreases with age achieving a local minimum around the age of sexual maturity. Thermodynamically, this highlights the system’s stability as a function of time. From that age forward, the Gibbs free energy slowly increases in almost all brain regions. Analogous findings have been reported by Yao et al. [13] who used entropy values to characterize intrinsic aging properties of the human brain from fMRI data sets. While we offer no concrete explanation of this effect, we hypothesize that this may reflect the brain’s architectural remodelling. Finally, we have also compared the Gibbs free energy for male and female samples, respectively. In most brain regions and also for the averaged values over all regions of the brain, we found that from prenatal stages of development until early childhood the Gibbs free energy from female subjects is higher than that of the male counterparts, which we interpret as accelerated development. According to literature, the brain metabolic demand (energy use) peaks during childhood [1]. A cross-over effect can be seen in early childhood where the relationship is reversed between female and male values of the Gibbs free energy, but eventually, around the age of 40, both curves converge to the same value. Admittedly, the relatively small sample size suggests that these results should be interpreted with great caution. In particular the dataset used has a gap between ages 4–8 years, which is the age when brain metabolism is at its peak. Thus, it is crucial that future studies fill in that age gap in order to remove this limitation and strengthen the importance of our findings. Larger sets of similar data would offer greater confidence in our analysis, but due to lack of such datasets, we are unable to provide more statistically significant conclusions at present.

Finally, it is worth providing a comment on the possible interpretation of the use of thermodynamic concepts within developmental neuroscience. Gibbs free energy, *G*, as often used in thermodynamics has another definition, namely:

*G* = *H* − *T S* (5)

where *H* denotes enthalpy, *T* the absolute temperature in degrees Kelvin, and *S* is entropy. Thermodynamic systems tend to equilibrium by minimizing their Gibbs free energy. As the above formula indicates, this can be accomplished by either minimizing enthalpy or maximizing entropy, or both. Enthalpy minimization corresponds to reducing the energy of the system by favourable (attractive) interactions between components of the system. Entropy maximization is achieved by increasing disorder in the system. In the present context of PPI networks, we can interpret these two tendencies in the development and transformations of the human brain as it ages as follows. Early development is marked by growth and structure formation, which corresponds to the tendency away from thermodynamic equilibrium highlighted by order and organization that reduces entropy and hence increases the Gibbs free energy. Subsequent stabilization of the Gibbs free energy at young adulthood corresponds to the establishment of a thermodynamic equilibrium at that age. Finally, as advanced age is approached, the concomitant tendency toward the increase of the Gibbs free energy is most likely due to protein aggregation processes, which reduce entropy and may reflect a functional deterioration of this organ as is the case with virtually all neurodegenerative diseases whose hallmarks include amyloid aggregation, but not only; for example, pathologies such as tauopathy in Alzheimer’s disease also involve function-reducing tangles composed of MAP-tau. Hence, we hypothesize that Gibbs free energy increases, while seen both in early brain development and advanced age, are driven by different types of processes. In the former case, they represent healthy structure formation while in the latter pathological aggregation processes related to loss of function.

Finally, we wish to comment on the general perspective that our paper provides in the context of brain development. It is generally known that the gross anatomy of the brain undergoes major morphological changes in both prenatal and neonatal stages. We also know that subsequent evolution of neuronal connections takes places during the process of maturation and into adulthood correlated with learning and acquisition of skills. What our results indicate is that there is also continuous transformation taking place throughout the human lifespan, which involves protein–protein interaction networks inside neurons. In other words, from the largest to the smallest hierarchies of organization of the brain, these structures undergo profound changes affecting their complex architecture and functions. This opens a new avenue for investigations into how these hierarchical changes are interconnected. In particular, how protein–protein interaction networks in neurons influence the connectivity of neuronal networks in specific brain regions since our results indicate inter-regional variations.

## Figures and Tables

**Table 1:** Summary of the abbreviations used for the brain regions with their descriptions and main functions performed.

Abbreviation	Brain Region	Main Functions
OFC	orbital prefrontal cortex	Reasoning, language, decision making
DFC	dorsolateral prefrontal cortex	Working memory, motor planning, abstract reasoning
VFC	ventrolateral prefrontal cortex	Decision making, regulation of emotions, flexible behavior
MFC	medial prefrontal cortex	Sensory motor processes, cognitive and affective processes
M1C	primary motor (M1) cortex	Motor function coordination
S1C	primary somatosensory (S1) cortex	Integration of afferent somatosensory inputs
IPC	posterior inferior parietal cortex	Language, mathematical operations, body image
A1C	primary auditory (A1) cortex	Auditory system
STC	posterior superior temporal cortex	Sensation of sound, processing of speech
ITC	inferior temporal cortex	Visual object recognition
V1C	primary visual (V1) cortex	Pattern recognition
HIP	hippocampus	Learning and memory
AMY	amygdala	Processing of emotions
STR	striatum	Motor and action planning, decision-making, motivation reinforcement
MD	mediodorsal nucleus of thalamus	Pain processing
CBC	cerebellar cortex	Sensory, motor, and association functions
IPC	posterior inferior parietal cortex	Language, mathematical operations, body image
A1C	primary auditory (A1) cortex	Auditory system
STC	posterior superior temporal cortex	Sensation of sound, processing of speech

## Supplementary Materials

Supplementary materials can be found at https://www.mdpi.com/1422-0067/21/3/1116/s1.
