# Supplementary material for: Using the Gibbs Function as a Measure of Human Brain Development Trends from Fetal Stage to Advanced Age
Source: Int J Mol Sci. 2020 Feb 7;21(3):1116. doi: 10.3390/ijms21031116 (PMC7037634; doi:10.3390/ijms21031116)
Supplement: Supplementary file 1 [file ijms-21-01116-s001.zip › SI-Figures/Supplementary Information.docx]

**Title: Thermodynamic Measures of Human Brain Development**

**from Fetal Stage to Adulthood**

Edward A. Rietman^1^, Sophie Taylor^2^, Hava T. Siegelmann^1^,

Marco Cavaglia^3^ and Jack A. Tuszynski^2,4,5^

1. BINDS Lab, School of Computer Science, University of Massachusetts Amherst, 140 Governors Drive, Amherst, MA, 01003-9264, United States
2. Department of Physics, University of Alberta, 4-181 CCIS, Edmonton, AB, T6G 2E1, Canada
3. ACTISMED, srl, Torino, Italy
4. DIMEAS, Politecnico di Torino, Corso Duca degli Abruzzi 24, Torino, 10129, Italy
5. Department of Oncology, University of Alberta, Cross Cancer Institute, 11560 University Avenue, Edmonton, AB, T6G 1Z2, Canada

**Supplementary Information**

In this Supplementary Information document we provide figures showing comparisons between make and female subjects according to each brain area.

For the **primary auditory cortex** A1C see Figure S1. For the **amygdala** AMY see Figure S2.

The data for the **cerebellar cortex** CBC is shown in Figure S3. The data for the **dorsolateral prefrontal cortex** DFC is shown in Figure S4. The **hippocampus** HIP data are illustrated in Figure S5. The data for the **posterior inferior parietal cortex** IPC can be seen in Figure S6.

For the fetal period for the **inferior temporal cortex** ITC see Figure S7. The Gibbs values for the **primary motor cortex** M1C are shown in Figure S8. The Gibbs values for the **mediodorsal nucleus** of the thalamus MD are given in Figure S9. For the **medial prefrontal cortex** MFC data, see Figure S10. The **orbital prefrontal cortex** OFC are shown in Figure S11. The Gibbs values for the **primary somatosensory cortex** S1C are presented in Figure S12. The **posterior superior temporal cortex** STC data are shown in Figure S13. For the **striatum** STR data, see Figure S14. The data for the **primary visual cortex** V1C are shown in Figure S15. For the data regarding the **ventrolateral prefrontal cortex** VFC, see Figure S16.

**Figure Legends**

**Figure S1.** Gibbs free energy for male and female samples in the primary auditory (A1C) cortex.

**Figure S2***.* Gibbs free energy for male and female samples in the amygdala (AMY).

**Figure S3**. Gibbs free energy for male and female samples in the cerebellar cortex (CBC).

**Figure S4.** Gibbs free energy for male and female samples in the dorsolateral prefrontal cortex (DFC)*.*

**Figure S5.** Gibbs free energy for male and female samples in the hippocampus (HIP).

**Figure S6.** Gibbs free energy for male and female samples in the posterior inferior parietal cortex (IPC).

**Figure S7.** Gibbs free energy for male and female samples in the inferior temporal cortex (ITC).

**Figure S8.** Gibbs free energy for male and female samples in the primary motor (M1C) cortex.

**Figure S9**. Gibbs free energy for male and female samples in the mediodorsal nucleus (MD) of thalamus.

**Figure S10**. Gibbs free energy for male and female samples in the medial prefrontal cortex (MFC).

**Figure S11**. Gibbs free energy for male and female samples in the orbital prefrontal cortex (OFC).

**Figure S12.** Gibbs free energy for male and female samples in the primary somatosensory (S1C) cortex.

**Figure S13.** Gibbs free energy for male and female samples in the posterior superior temporal cortex (STC).

**Figure S14.** Gibbs free energy for male and female samples in the striatum (STR).

**Figure S15.** Gibbs free energy for male and female samples in the primary visual (V1C) cortex.

**Figure S16.** Gibbs free energy for male and female samples in the ventrolateral prefrontal cortex (VFC).
