# Supplementary figures and images for: Using the Gibbs Function as a Measure of Human Brain Development Trends from Fetal Stage to Advanced Age
Source: Int J Mol Sci. 2020 Feb 7;21(3):1116. doi: 10.3390/ijms21031116 (PMC7037634; doi:10.3390/ijms21031116)

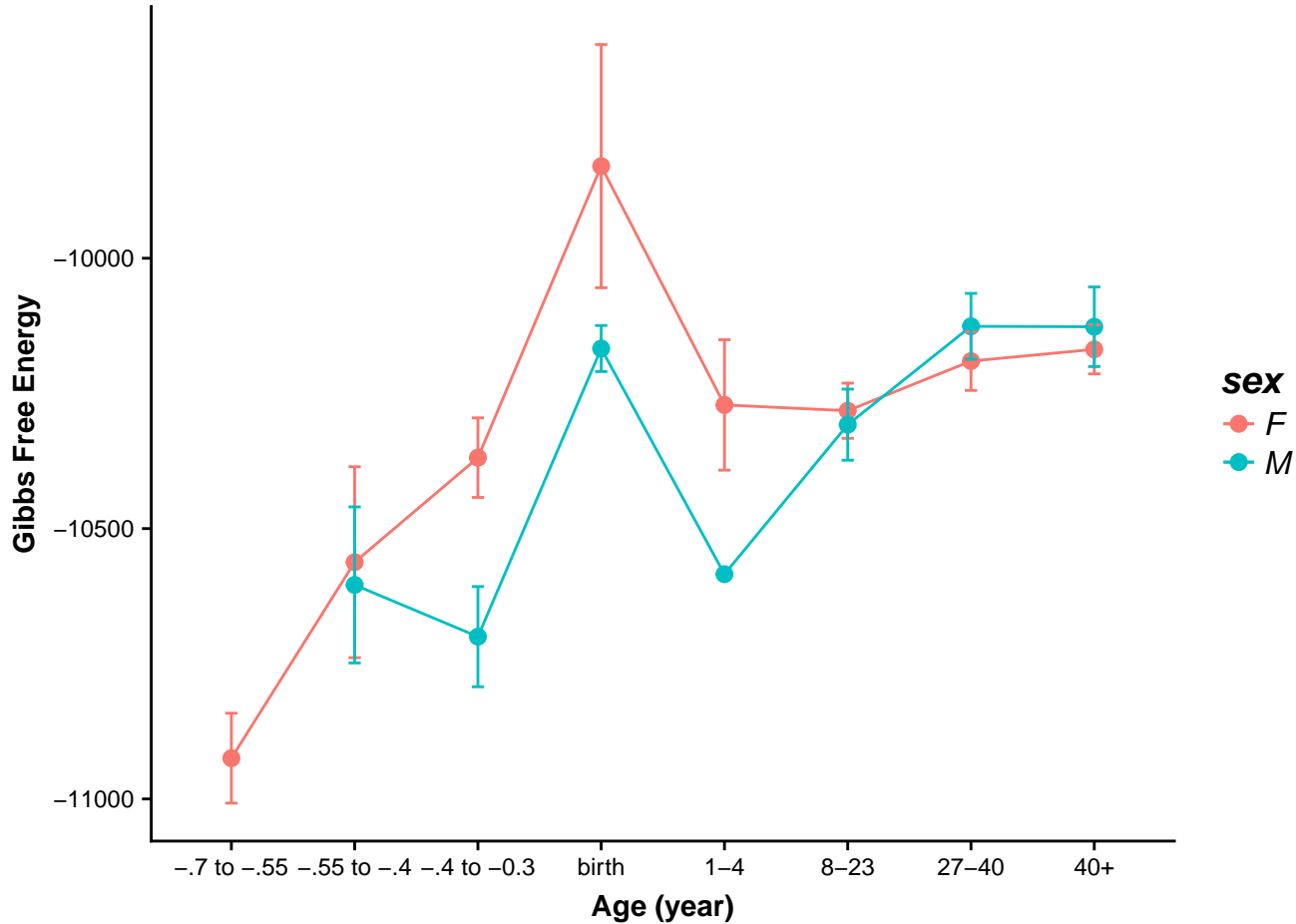

Supplement: Supplementary file 1 [file ijms-21-01116-s001.zip › SI-Figures/Fig.S9.pdf]

Gibbs Free Energy

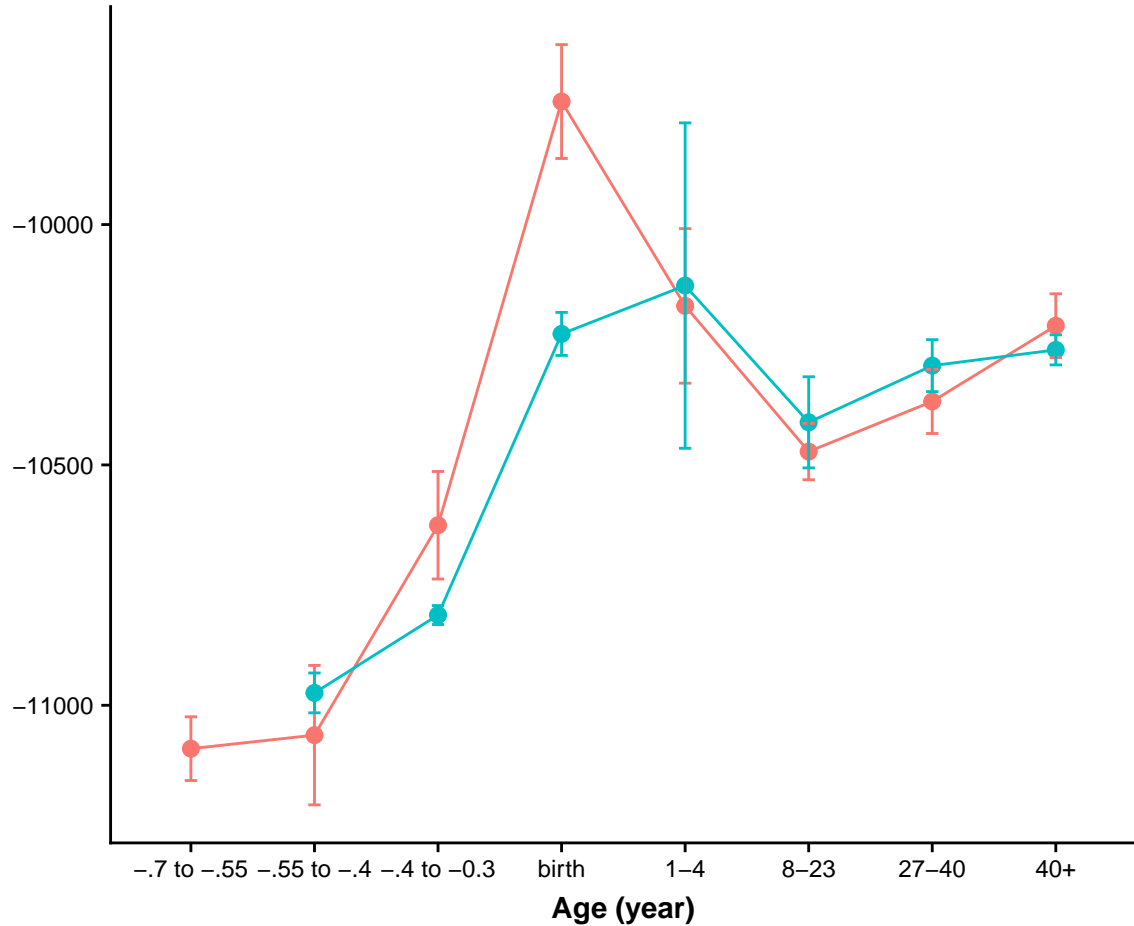

Supplement: Supplementary file 1 [file ijms-21-01116-s001.zip › SI-Figures/Fig.S8.pdf]

Gibbs Free Energy

-10000

-10400

-10800

-11200

-0.7 to -0.55 -0.55 to -0.4 -0.4 to -0.3 birth 1-4 8-23 27-40 40+

Age (year)

sex

F

M

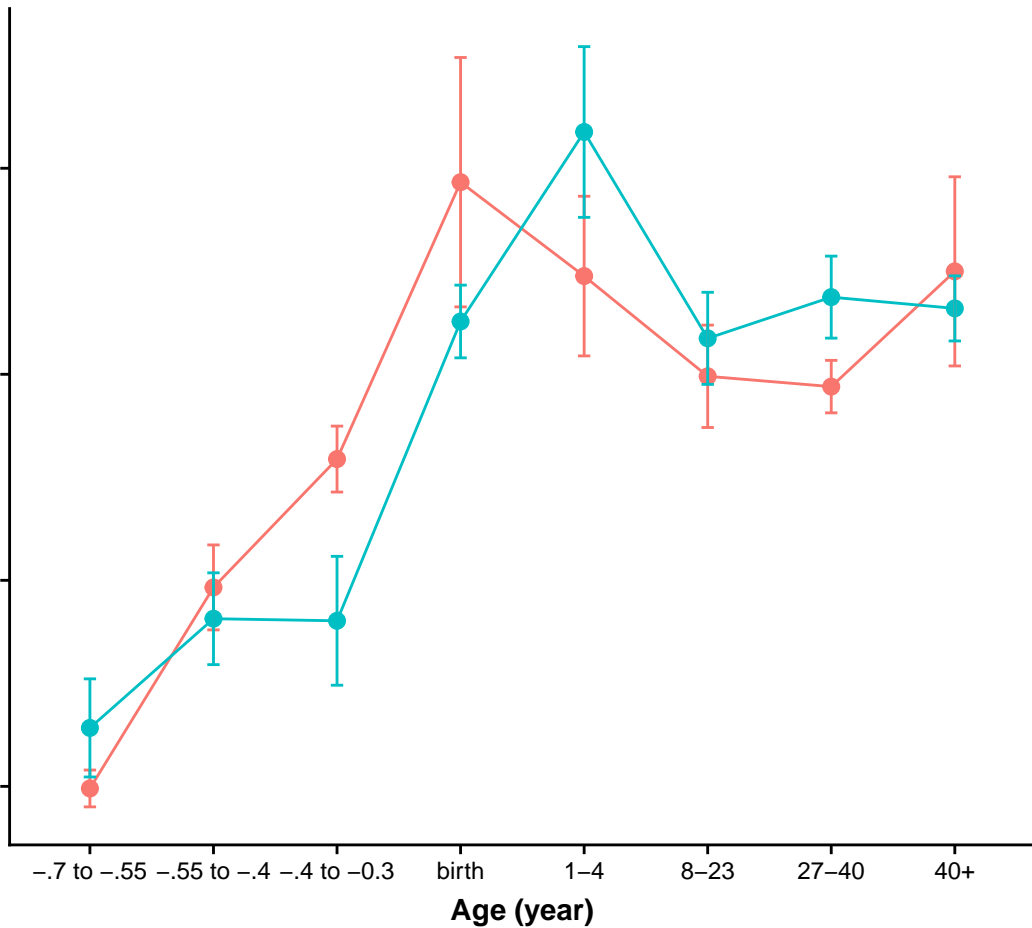

Supplement: Supplementary file 1 [file ijms-21-01116-s001.zip › SI-Figures/Fig.S13.pdf]

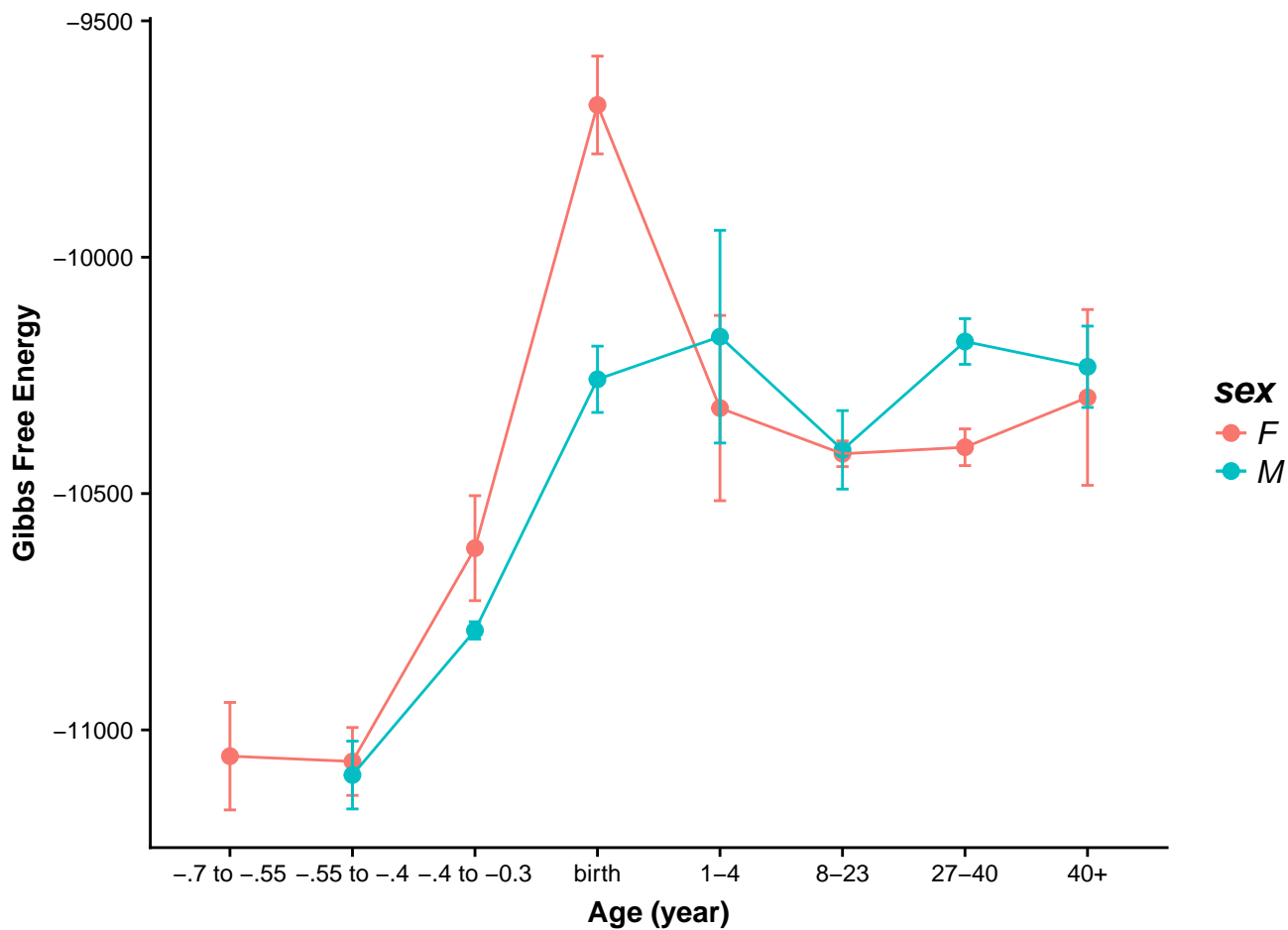

Supplement: Supplementary file 1 [file ijms-21-01116-s001.zip › SI-Figures/Fig.S12.pdf]

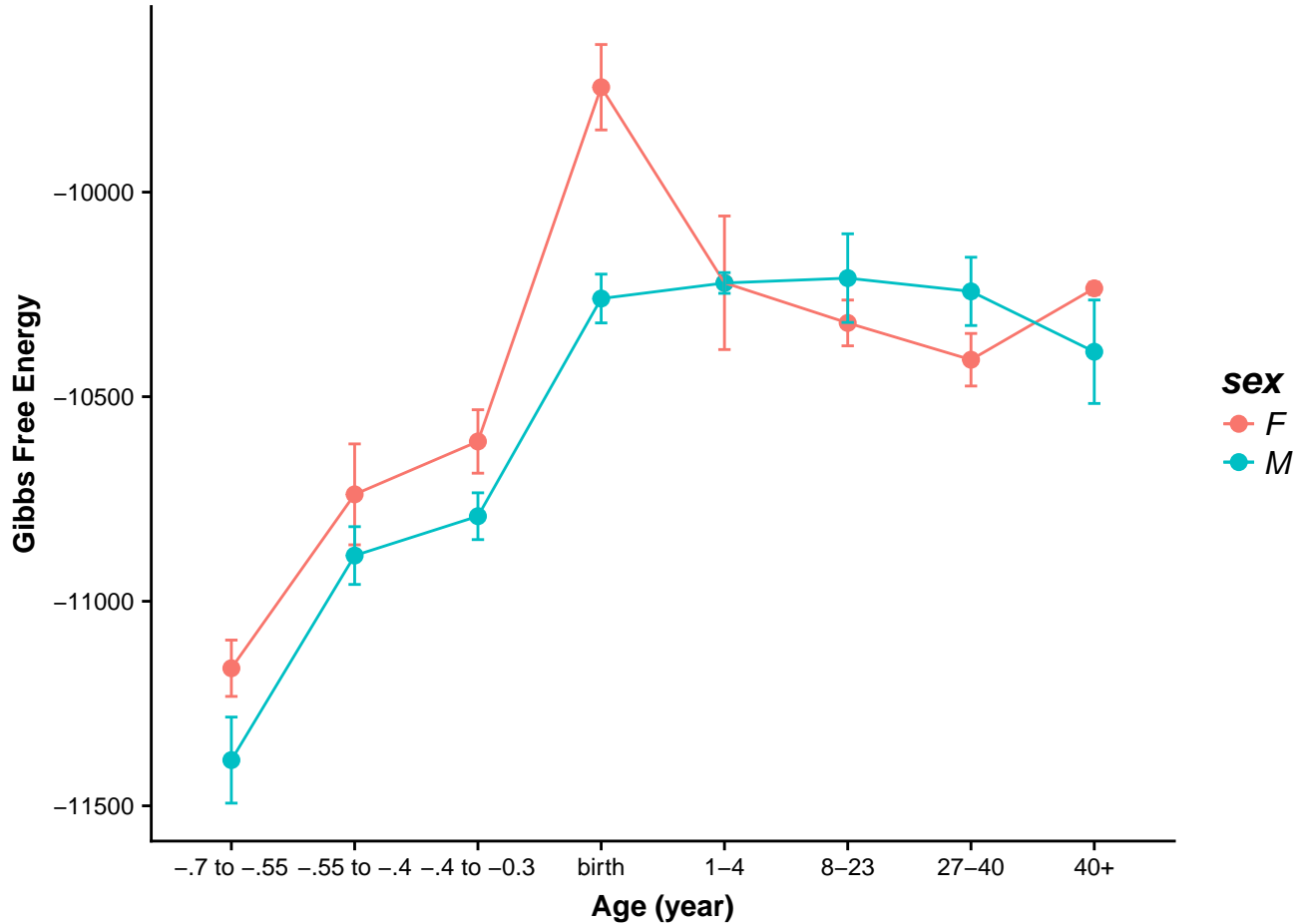

Supplement: Supplementary file 1 [file ijms-21-01116-s001.zip › SI-Figures/Fig.S10.pdf]

Gibbs Free Energy

-11200

-10800

-10400

-10000

-0.7 to -0.55   -0.55 to -0.4   -0.4 to -0.3   birth   1-4   8-23   27-40   40+

Age (year)

**sex**

*F*

*M*

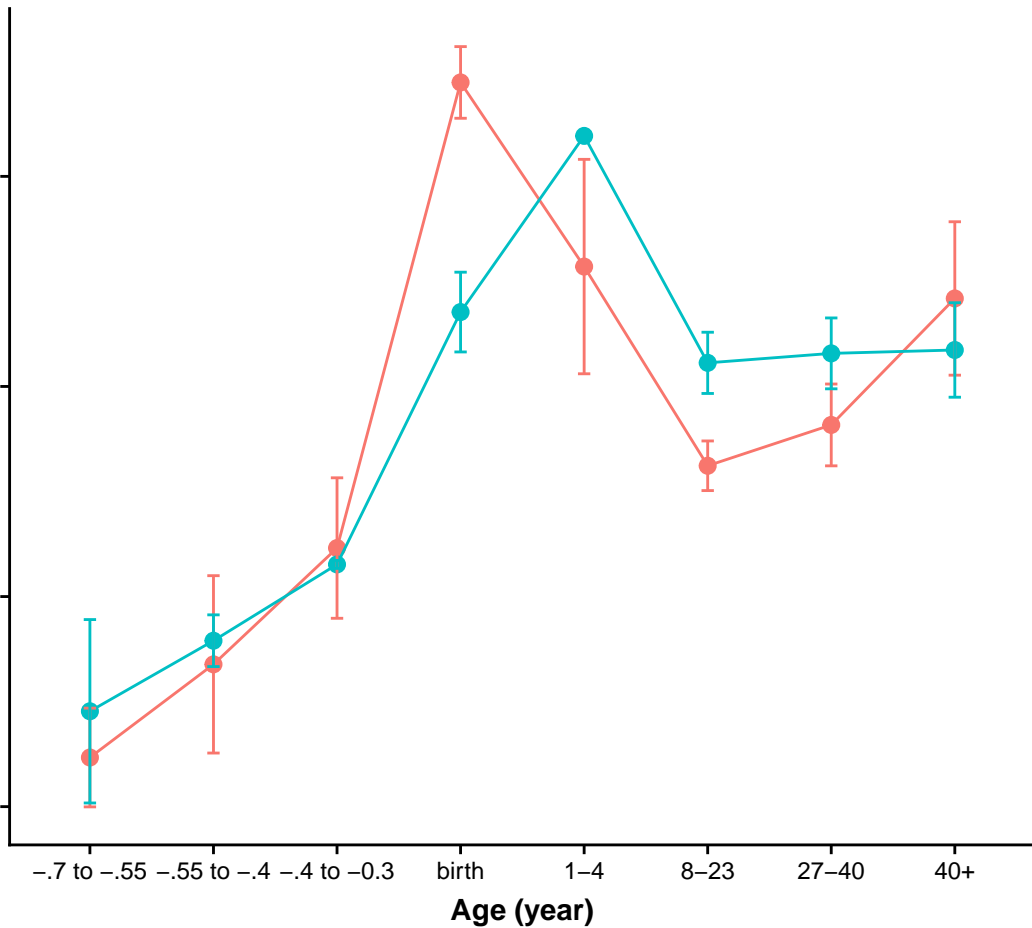

Supplement: Supplementary file 1 [file ijms-21-01116-s001.zip › SI-Figures/Fig.S11.pdf]

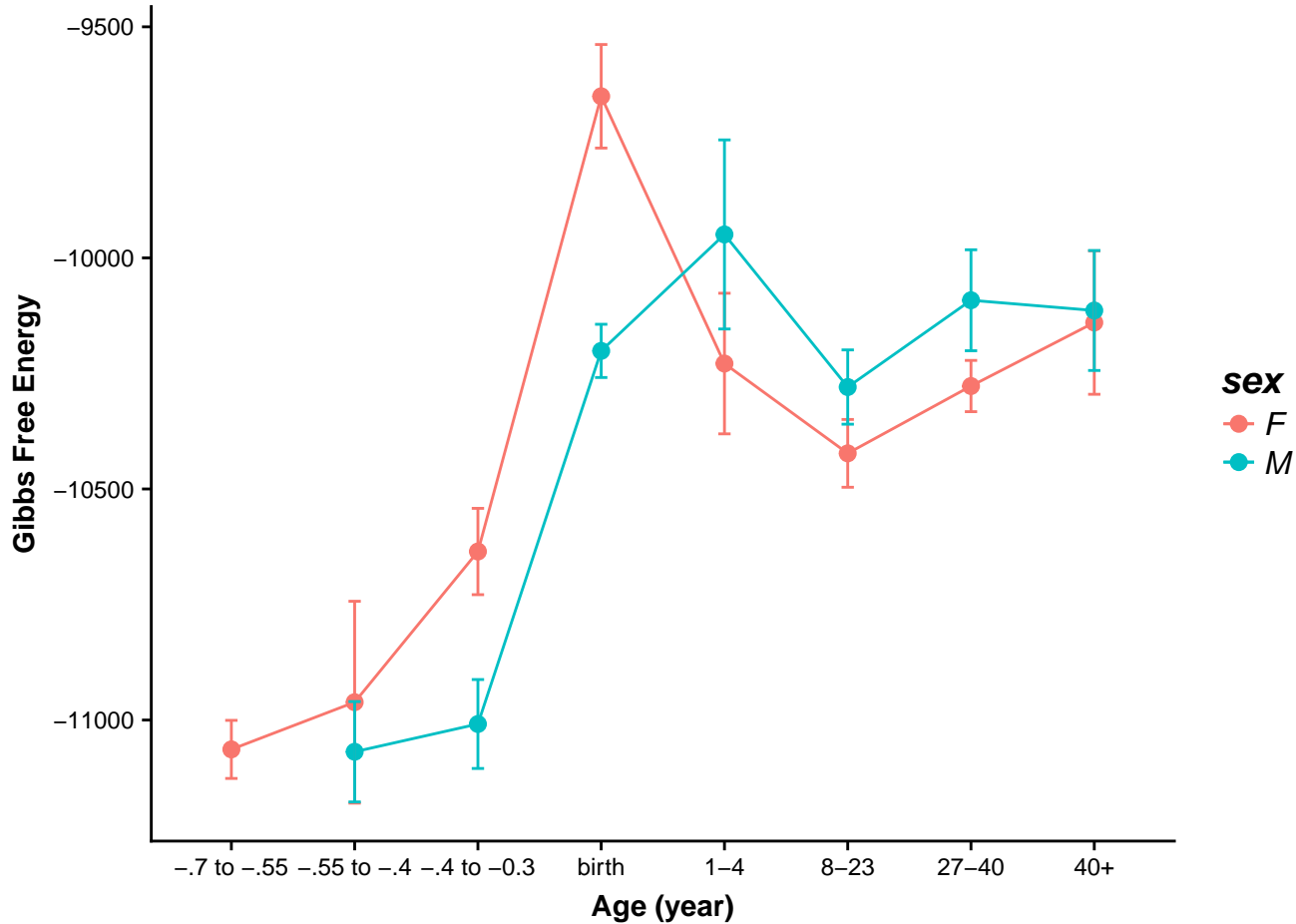

Supplement: Supplementary file 1 [file ijms-21-01116-s001.zip › SI-Figures/Fig.S15.pdf]

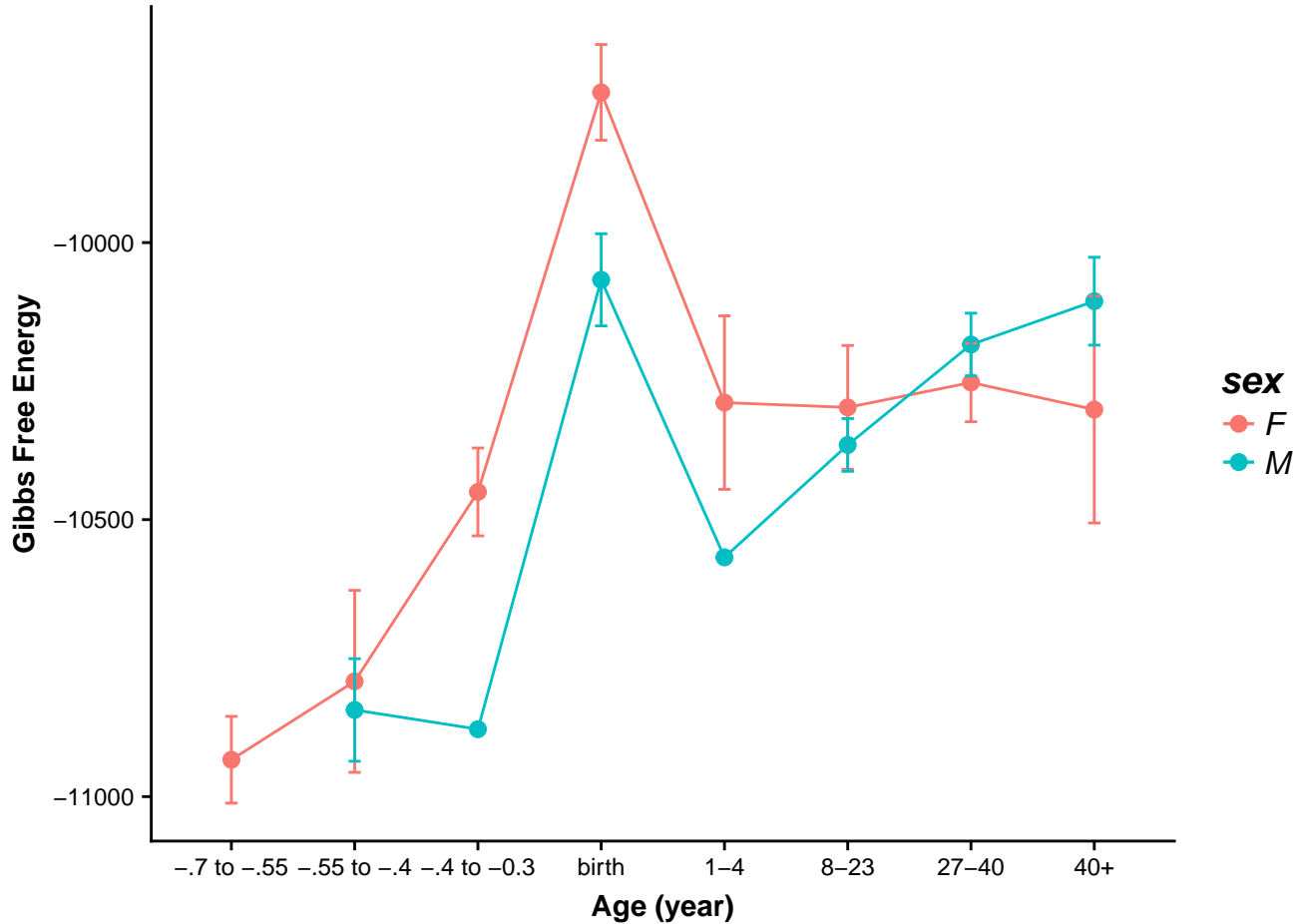

Supplement: Supplementary file 1 [file ijms-21-01116-s001.zip › SI-Figures/Fig.S14.pdf]

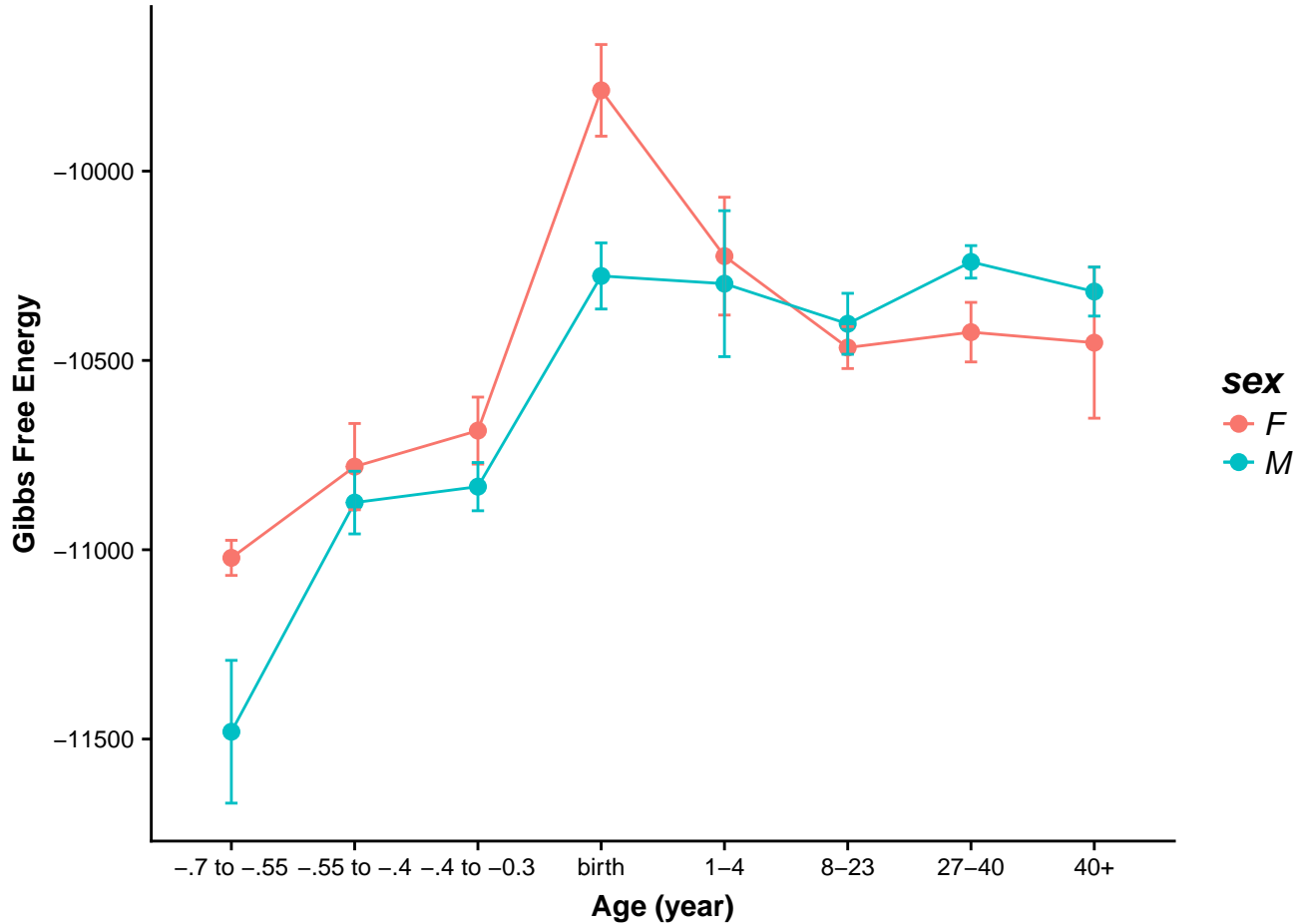

Supplement: Supplementary file 1 [file ijms-21-01116-s001.zip › SI-Figures/Fig.S16.pdf]

Gibbs Free Energy

-11000

-10500

-10000

-0.7 to -0.55   -0.55 to -0.4   -0.4 to -0.3   birth   1-4   8-23   27-40   40+

Age (year)

sex

F

M

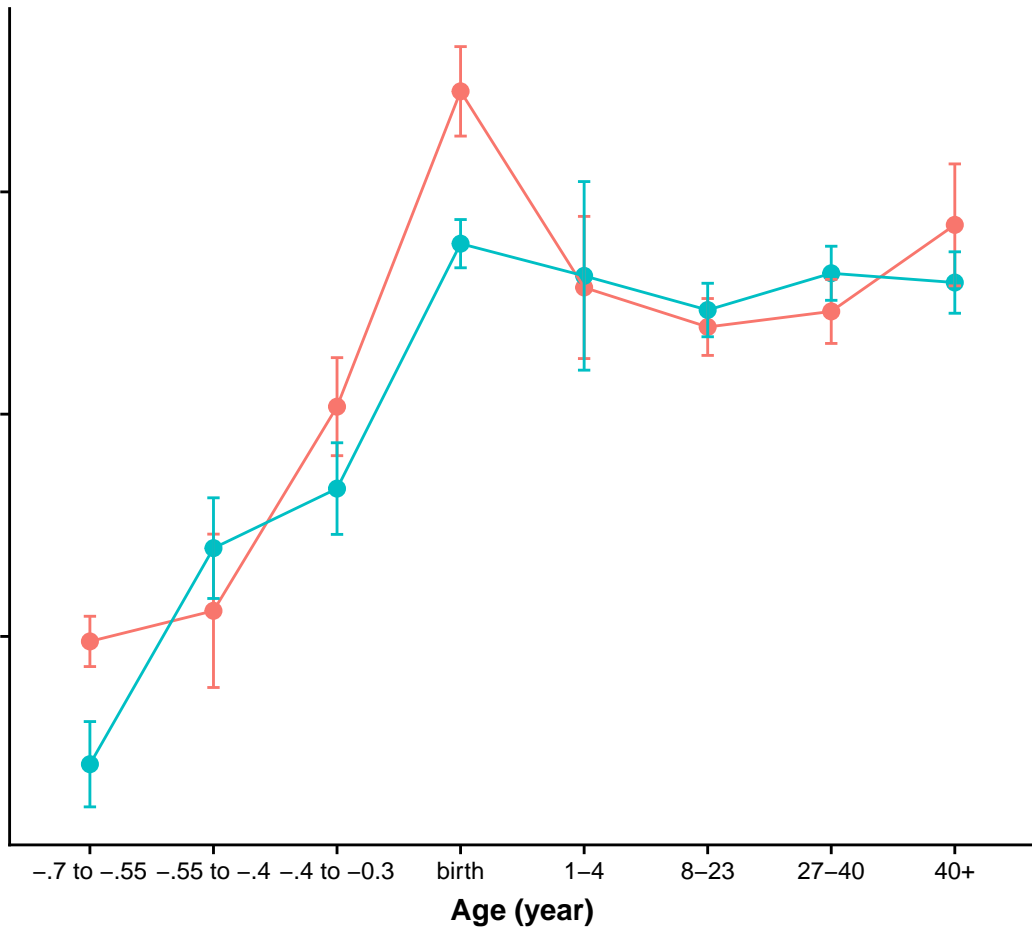

Supplement: Supplementary file 1 [file ijms-21-01116-s001.zip › SI-Figures/Fig.S5.pdf]

Gibbs Free Energy

-10000

-10500

-11000

-0.7 to -0.55   -0.55 to -0.4   -0.4 to -0.3   birth   1-4   8-23   27-40   40+

Age (year)

**sex**

*F*

*M*

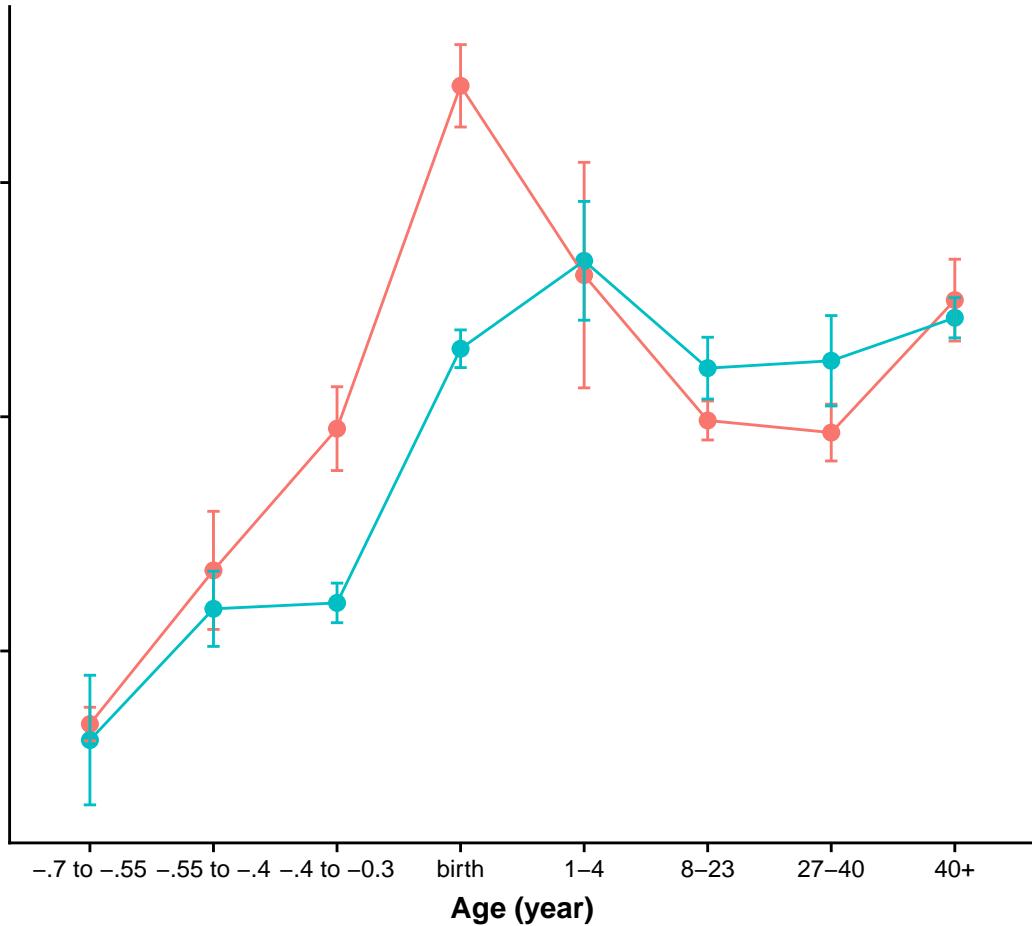

Supplement: Supplementary file 1 [file ijms-21-01116-s001.zip › SI-Figures/Fig.S4.pdf]

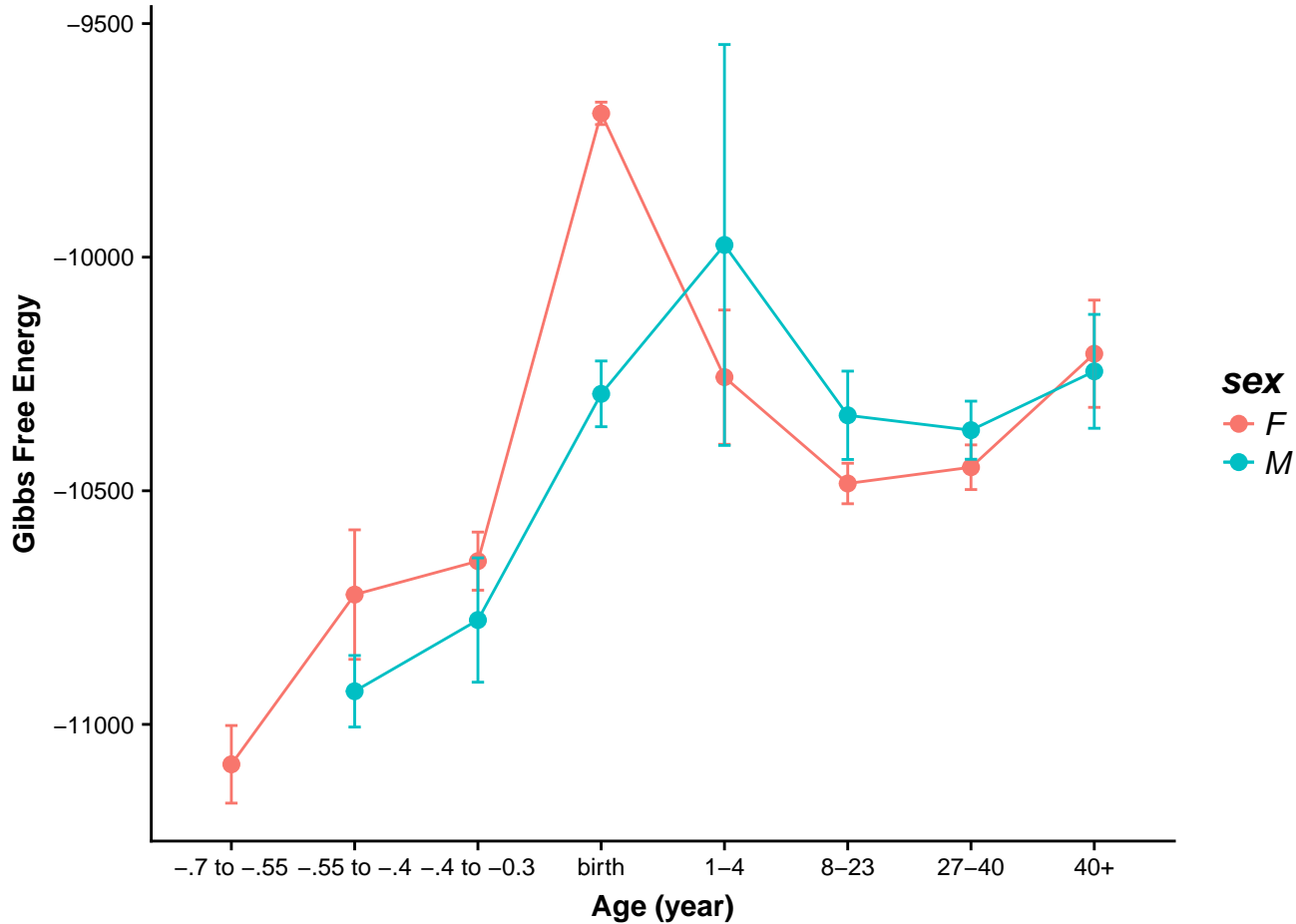

Supplement: Supplementary file 1 [file ijms-21-01116-s001.zip › SI-Figures/Fig.S6.pdf]

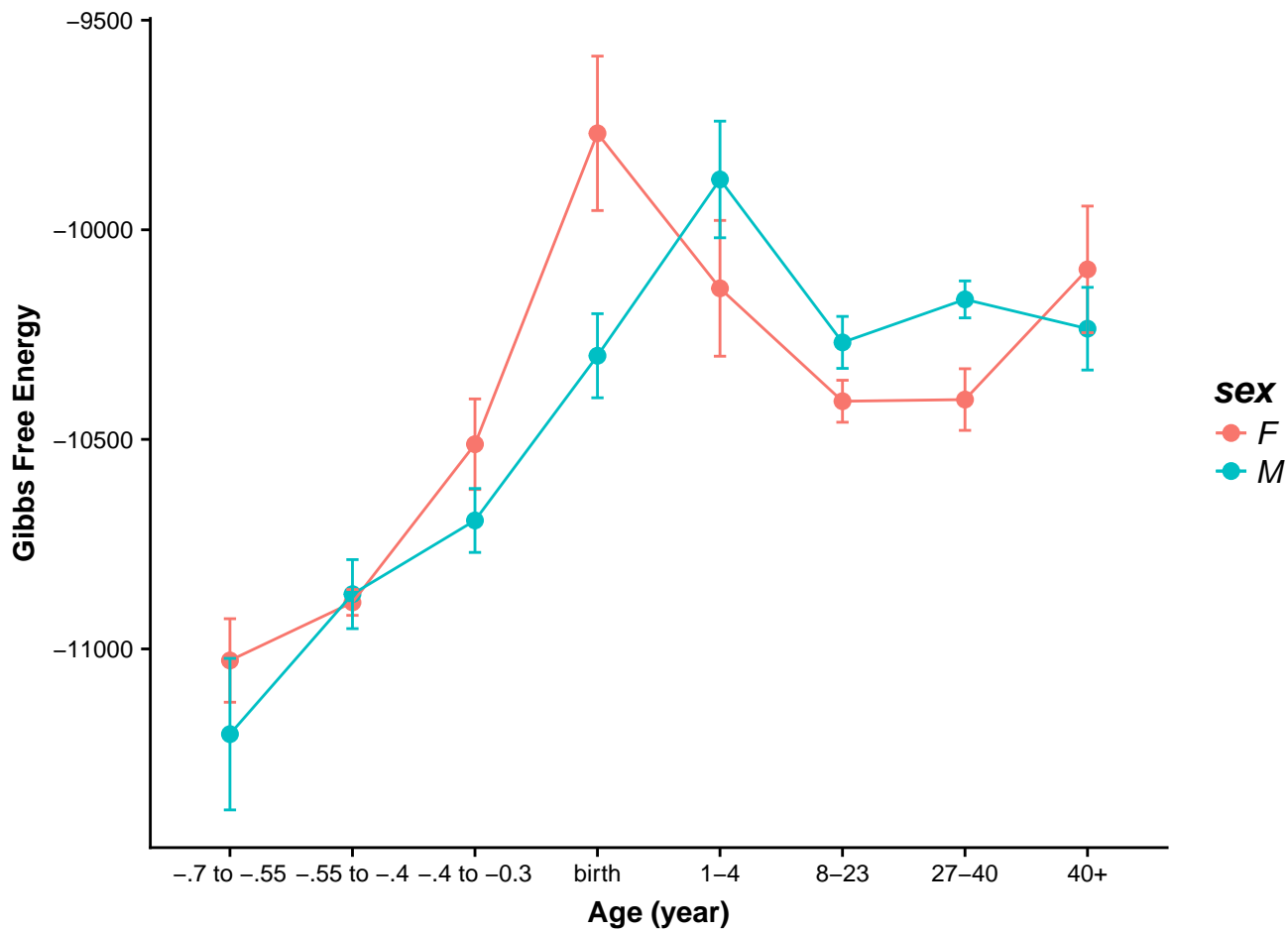

Supplement: Supplementary file 1 [file ijms-21-01116-s001.zip › SI-Figures/Fig.S7.pdf]

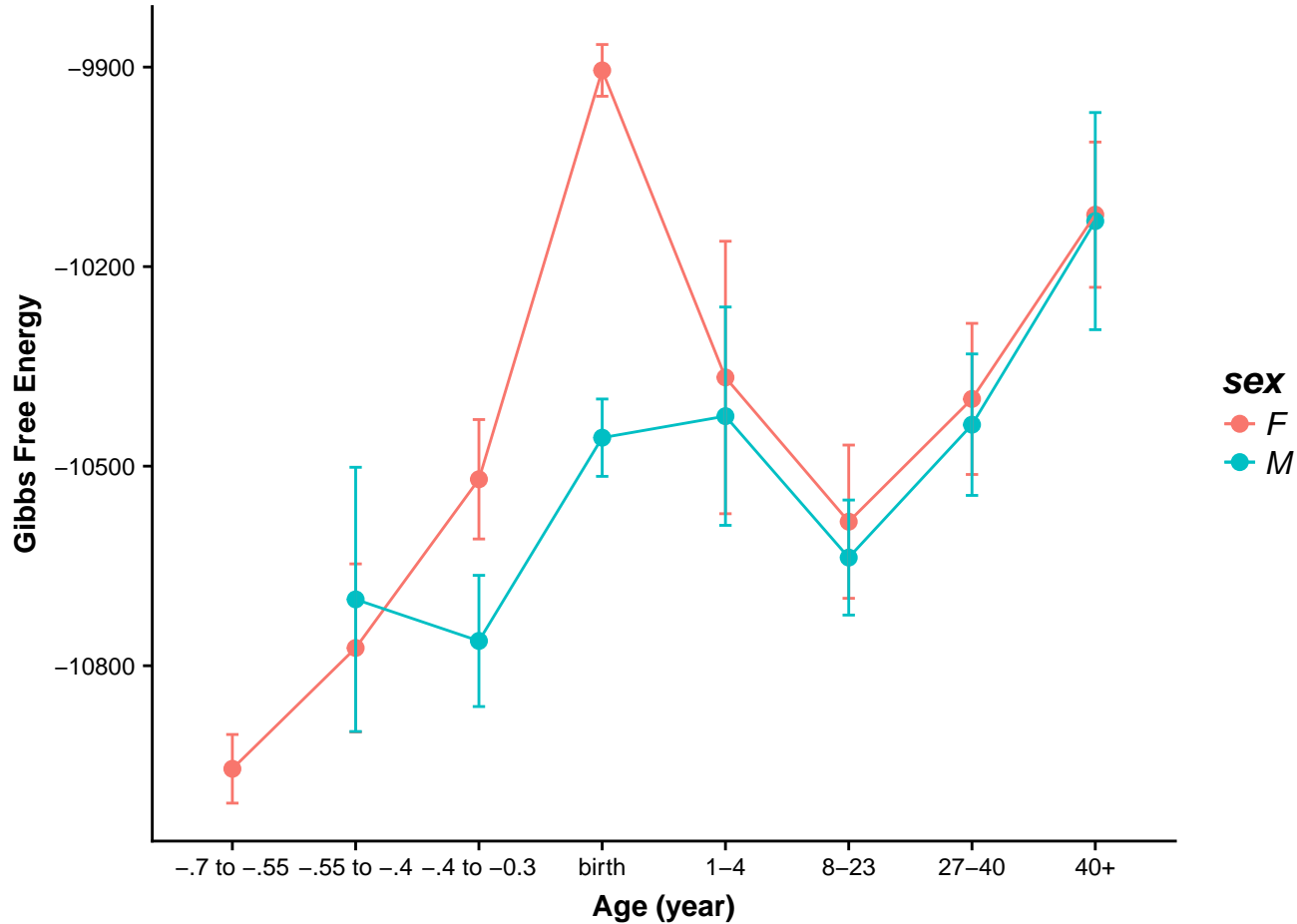

Supplement: Supplementary file 1 [file ijms-21-01116-s001.zip › SI-Figures/Fig.S3.pdf]

Gibbs Free Energy

Age (year)

**sex**

*F*

*M*

-10000

-10400

-10800

-11200

-0.7 to -0.55

-0.55 to -0.4

-0.4 to -0.3

birth

1-4

8-23

27-40

40+

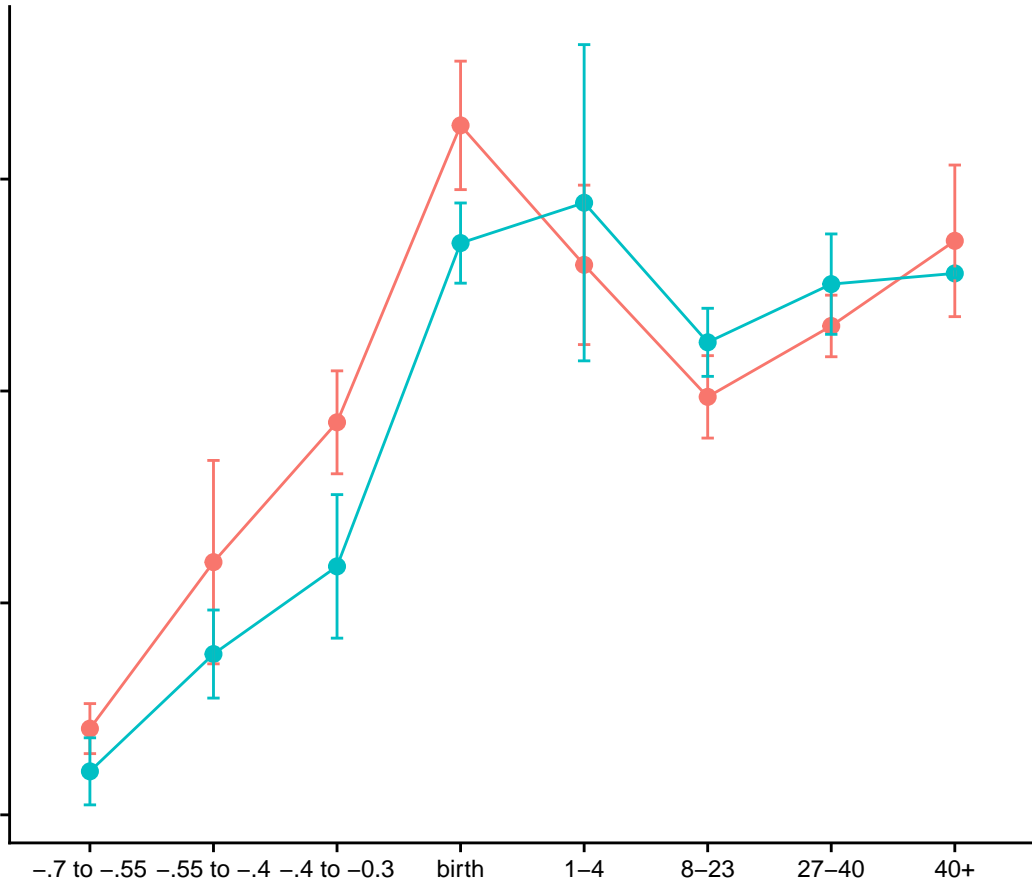

Supplement: Supplementary file 1 [file ijms-21-01116-s001.zip › SI-Figures/Fig.S2.pdf]

Gibbs Free Energy

-11200  
-10800  
-10400  
-10000

-0.7 to -0.55   -0.55 to -0.4   -0.4 to -0.3   birth   1-4   8-23   27-40   40+

Age (year)

**sex**

*F*

*M*

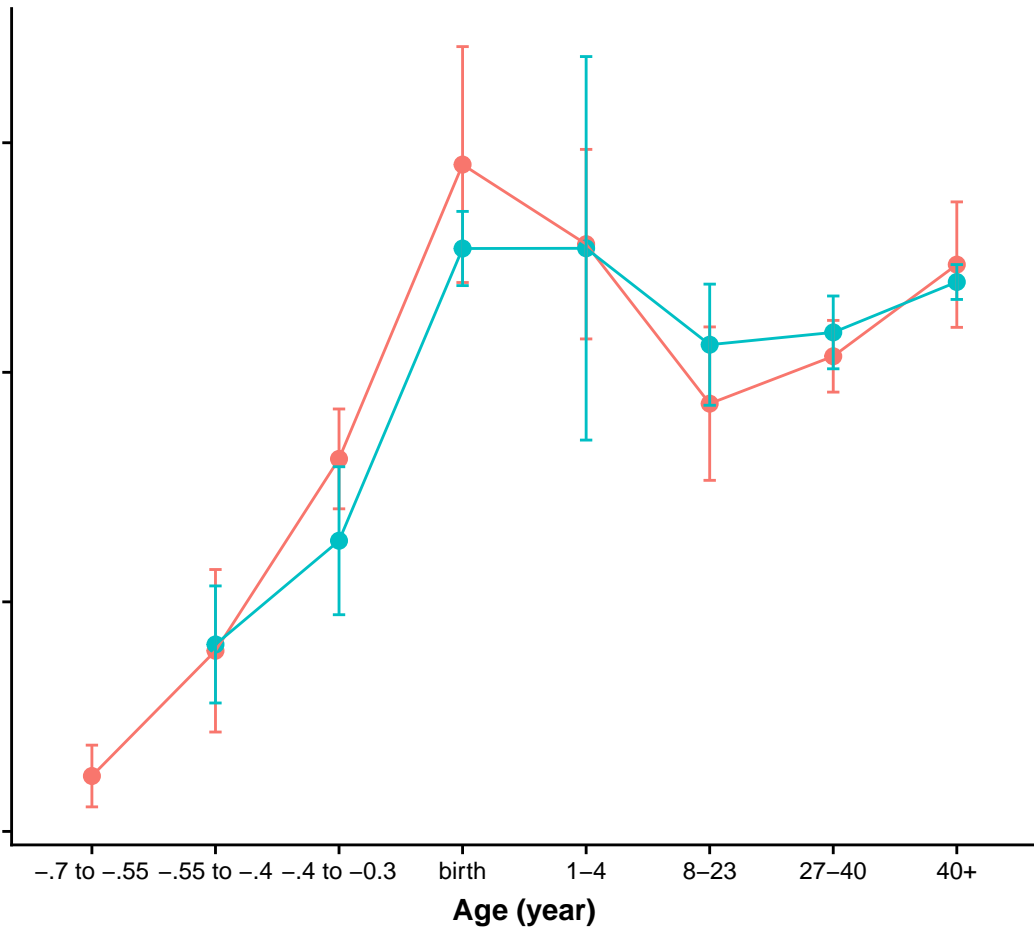

Supplement: Supplementary file 1 [file ijms-21-01116-s001.zip › SI-Figures/Fig.S1.pdf]
